# Paediatric surgical outcomes in sub-Saharan Africa: a multicentre, international, prospective cohort study

**DOI:** 10.1136/bmjgh-2020-004406

**Published:** 2021-09-02

**Authors:** PaedSurg Africa Research Collaboration

**Keywords:** paediatrics, surgery, child health, epidemiology, treatment

## Abstract

**Introduction:**

As childhood mortality from infectious diseases falls across sub-Saharan Africa (SSA), the burden of disease attributed to surgical conditions is increasing. However, limited data exist on paediatric surgical outcomes in SSA. We compared the outcomes of five common paediatric surgical conditions in SSA with published benchmark data from high-income countries (HICs).

**Methods:**

A multicentre, international, prospective cohort study was undertaken in hospitals providing paediatric surgical care across SSA. Data were collected on consecutive children (birth to 16 years), presenting with gastroschisis, anorectal malformation, intussusception, appendicitis or inguinal hernia, over a minimum of 1 month, between October 2016 and April 2017. Participating hospitals completed a survey on their resources available for paediatric surgery.

The primary outcome was all-cause in-hospital mortality. Mortality in SSA was compared with published benchmark mortality in HICs using χ^2^ analysis. Generalised linear mixed models were used to identify patient-level and hospital-level factors affecting mortality. A p<0.05 was deemed significant.

**Results:**

1407 children from 51 hospitals in 19 countries across SSA were studied: 111 with gastroschisis, 188 anorectal malformation, 225 intussusception, 250 appendicitis and 633 inguinal hernia. Mortality was significantly higher in SSA compared with HICs for all conditions: gastroschisis (75.5% vs 2.0%), anorectal malformation (11.2% vs 2.9%), intussusception (9.4% vs 0.2%), appendicitis (0.4% vs 0.0%) and inguinal hernia (0.2% vs 0.0%), respectively. Mortality was 41.9% (112/267) among neonates, 5.0% (20/403) in infants and 1.0% (7/720) in children. Paediatric surgical condition, higher American Society of Anesthesiologists score at primary intervention, and needing/receiving a blood transfusion were significantly associated with mortality on multivariable analysis.

**Conclusion:**

Mortality from common paediatric surgical conditions is unacceptably high in SSA compared with HICs, particularly for neonates. Interventions to reduce mortality should focus on improving resuscitation and timely transfer at the district level, and preoperative resuscitation and perioperative care at paediatric surgical centres.

**Key questions**

**What is already known?**

- 1.7 billion children worldwide do not have access to safe, affordable, timely surgical care when needed.
- Emergency abdominal surgical outcomes in children are poor in low-income and middle-income countries, compared with high-income countries (HICs), globally.
- Sub-Saharan Africa (SSA), where up to 50% of the population are children, has the greatest burden of surgical disease, but limited data exist on paediatric surgical management and outcomes in the region.

**What are the new findings?**

- In a prospective cohort study of 1407 children presenting to 51 hospitals in 19 low-income and middle-income countries across SSA, the mortality from common paediatric surgical conditions was significantly higher than reported in HICs.
- The majority of deaths occurred in neonates.
- Mortality was associated with indicators of poor systemic condition at presentation to the paediatric surgery centre and at the time of primary intervention.

**What do the new findings imply?**

- Mortality from common paediatric surgical conditions is unacceptably high in SSA compared with HICs, particularly for neonates.
- Sustainable Development Goal 3.2 to ‘end preventable deaths in neonates and children under 5 by 2030’ is unachievable without urgent action to improve access to quality paediatric surgical care in SSA.
- Interventions to reduce mortality should focus on improving resuscitation and timely transfer at the district level, and preoperative resuscitation and perioperative care at paediatric surgical centres.

## Introduction

The global focus on reducing childhood mortality from infectious diseases is reflected in improved outcomes.^1^ However, lack of focus on surgical diseases in children means the proportion of deaths attributed to surgical conditions is increasing.^1–3^ Globally, 1.7 billion children do not have access to safe, affordable, timely surgical care when needed.^2^ Sub-Saharan Africa (SSA), where up to 50% of the population are children, has almost one-third of the worlds burden of surgical disease and half of the worlds under-5 deaths.^4–6^ A population-based study in Uganda highlighted that almost one-third of childhood deaths resulted from surgical conditions.^7^ Current evidence from SSA on paediatric surgical outcomes comes mainly from single-institutions, small case series and retrospective reviews. Multicentre, multinational prospective data are lacking.^8^

This study sought to address this gap by collecting prospective data from hospitals providing paediatric surgical care across SSA on five of the most common paediatric surgical conditions in the neonatal, infant and childhood periods: gastroschisis, anorectal malformation (ARM), intussusception, appendicitis and inguinal hernia. Gastroschisis is one of the most common congenital anomalies with a rising global incidence. An international survey estimated many paediatric surgical centres in SSA have a mortality of 75%–100% from gastroschisis, compared with under 25% in high-income countries (HICs).^9–12^ Associated anomalies are rare in neonates with this condition and in HICs the vast majority live a full life with minimum disability. ARM, also known as imperforate anus, is one of the most frequent neonatal surgical emergencies presenting to hospitals in SSA with a mortality up to 20% in Nigeria compared with 3% in HICs.^13–15^ Intussusception is the leading cause of intestinal obstruction in children, typically infants, with reported mortality rates of 9.4% in SSA compared with 0.1% in Europe.^16^ Appendicitis is one of the the most common causes of acute abdomen in children worldwide. Research from SSA suggests deaths (extremely rare in HICs) still occur, and morbidity is high.^17–19^ Inguinal hernia repair is the most commonly performed paediatric operation worldwide; morbidity is low in HICs and mortality close to nil.^20^ Reports from SSA suggest delayed treatment and limited care facilities result in preventable deaths and disability.^21^

The study aim was to collect mortality and morbidity outcomes data for neonates and children presenting with these common paediatric surgical conditions to paediatric surgical centres across SSA, to compare mortality with published HIC benchmark data, and to identify factors associated with mortality that could be modified to improve survival.

## Methods

### Study design and participants

A multicentre, international prospective cohort study was undertaken at paediatric surgery centres across SSA. Healthcare professionals involved in the care of children with the study conditions in SSA, including surgeons, anaesthetists and allied health professionals, were invited to participate in the study through professional organisations, conference presentations, social media and designated ‘Country Leads’. At each participating hospital, data was collected by one or more teams, consisting of up to three local investigators per team, over 1-month or multiple 1-month study periods between October 2016 and April 2017. During each month of study participation, prospective data were collected on consecutive patients, under the age of 16 years, presenting for the first time with gastroschisis, ARM, intussusception, appendicitis and inguinal hernia. This included patients managed operatively and non-operatively for gastroschisis, ARM, intussusception and appendicitis. Children with an inguinal hernia required an operation to be included because, unlike the other conditions, patients can present electively and surgery is the only definitive intervention. Patients who had previously been operated and re-presented with complications or requiring further surgery were excluded.

Data were collected on patient demographics, condition on arrival, perioperative resuscitation and care, surgical intervention and outcomes (online supplemental appendix methods 1) using Research Electronic Data Capture (REDCap).^22^ Data collection consisted of generic variables, relevant to all the study conditions, and condition specific variables. Patients were followed up until discharge and 30 days post-intervention where locally feasible. The study protocol and data collection form were available in English, French and Portuguese. A pilot study was undertaken at five hospitals to optimise the study design and data collection. Data entered into REDCap was assessed for congruency and completeness. Local investigators were individually contacted for clarification of any discrepancies, and to complete missing data.

10.1136/bmjgh-2020-004406.supp1 Supplementary data

Local investigators were asked to complete a survey on the resources available for paediatric surgery at their hospital. This contained four categories: (1) personnel, (2) infrastructure, (3) procedures, (4) anaesthesia and resuscitation (online supplemental appendix methods 2). This was developed through modification of the PediPIPES institutional capacity assessment survey with a reduced number of questions for feasibility and to reflect the conditions being studied.^23^ ^24^

### Outcomes

The primary outcome was all-cause in-hospital mortality. For patients still in hospital, a 30-day post primary intervention mortality was used (including operative and non-operative interventions). Secondary outcomes included surgical site infection, wound dehiscence, need for unplanned re-intervention, length of hospital stay and 30-day post primary intervention mortality.

### Statistical analysis

We aimed to include a minimum of 50 hospitals (online supplemental appendix methods 3). Differences between all-cause, in-hospital mortality in SSA and expected mortality from published HIC benchmark data were compared using χ^2^ analysis or Fisher’s exact test if less than five patients per group. Results are presented with 95% CI. HIC benchmark studies were selected based on the most representative population and largest sample size at the time of protocol development (online supplemental appendix table 1).^10 15 16 20 25^ HIC gastroschisis mortality of 2.0% (6/302) within the neonatal period, was reported in a UK national ‘whole population’ cohort study.^10^ ARM HIC mortality of 2.9% (12/410) was reported from a case-series in Finland (270 high ARM; 140 low ARM).^15^ The HIC intussusception mortality of 0.2% (18/9186) constitutes a meta-analysis of ‘case-fatality among children hospitalised with intussusception’ in Australia, Europe, New Zealand and USA.^16^ Paediatric appendicitis mortality of 0.004% (1/24 665) in HICs, is from a meta-analysis of three hospital based studies in Canada, UK and USA containing both paediatric and general surgeon operators.^25^ HIC paediatric inguinal hernia mortality of 0% (0/6361) was reported from a 35-year case-series in Canada.^20^

A generalised linear mixed model (GLMM) with a binomial family specification and random intercept for hospitals was used to identify patient-level factors associated with all-cause in-hospital mortality for the three study conditions with highest mortality: gastroschisis, ARM and intussusception. A second GLMM was used to identify both patient-level and hospital-level factors associated with mortality for these three conditions combined. Variables significantly associated with mortality at univariable analysis (p<0.05) were included in the multivariable models, with adjustment for confounders: paediatric surgical condition, weight and American Society of Anesthesiologists (ASA) score. Exploratory analyses involved univariable analysis (including a random intercept for hospitals) of factors associated with all-cause, in-hospital mortality for gastroschisis, ARM and intussusception, individually. Multivariable analysis was not possible for the individual conditions due to low event rates per variable.

To facilitate analysis, hospital variables within each of the four domains (personnel, infrastructure, procedures and anaesthesia/resuscitation) were categorised into low, medium or high availability. For each hospital, the number of paediatric surgeons per population of children was calculated using the reported number of paediatric surgeons and population served in the hospital survey, and a published mean proportion of children in the SSA population of 43%.^26^ We used the IQR to categorise personnel availability as follows: low, <0.2 paediatric surgeons per 1 000 000 children; medium, ≥0.2 and <1.5 paediatric surgeons per 1 000 000 children; high, ≥1.5 paediatric surgeons per 1 000 000 children. For the infrastructure, procedures and anaesthesia/resuscitation domains, each variable was categorised as ‘Always available’ or ‘Sometimes or never available’. Each domain was then categorised as follows: low, indicating 30% or less of the variables within the domain were ‘always available’; medium, indicating between 31% and 69% of the variables within the domain were ‘always available’; high, indicating 70% or more of the variables within the domain were ‘always available’. Since a number of collaborators had independently completed the survey at each hospital, the mean for each variable was used for categorisation and analysis.

### Data validation

Ten per cent of study hospitals were randomly selected to validate patient data. A local investigator, who did not participate in the original data collection, but also cared for the study patients, was asked to independently collect data on all eligible patients presenting during 1 month of the study. Cohen’s kappa coefficient was used to evaluate the level of agreement between the main study and validation datasets. The validating investigator was asked to confirm how many eligible patients presented during the study period to determine if any were missed. A validation survey was undertaken by all local investigators at validating hospitals regarding the quality of the patient data. The hospital data were validated by analysing the level of agreement between local investigators independently completing the survey from the same hospital. Interclass correlation coefficients were used because the number of survey respondents per hospital was not consistent. Observed agreement was also reported.

Analyses were undertaken in Stata V.14, Stata V.15, R V.4.0 and SAS V.9.4. A p<0.05 was deemed significant.

### Patient and public involvement

Patients and public were not involved in the design of the study. However, patient support groups (One in 5000 Foundation and CDH International) are assisting in the dissemination of the findings.^27 28^

## Results

### Patient characteristics and exposures

**Figure 1:**
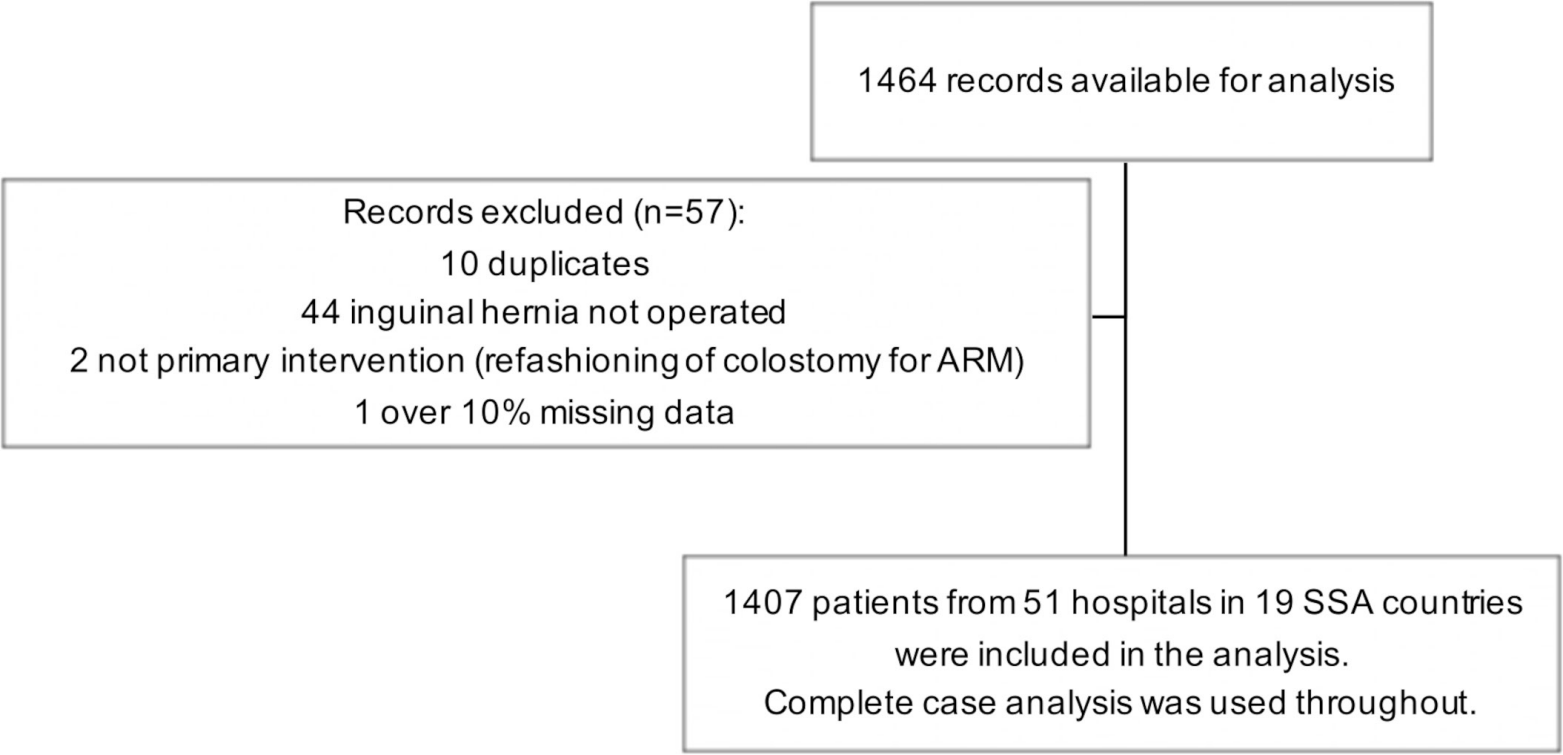
Flow chart of patient inclusion. ARM, anorectal malformation; SSA, sub-Saharan Africa.

**Figure 2:**
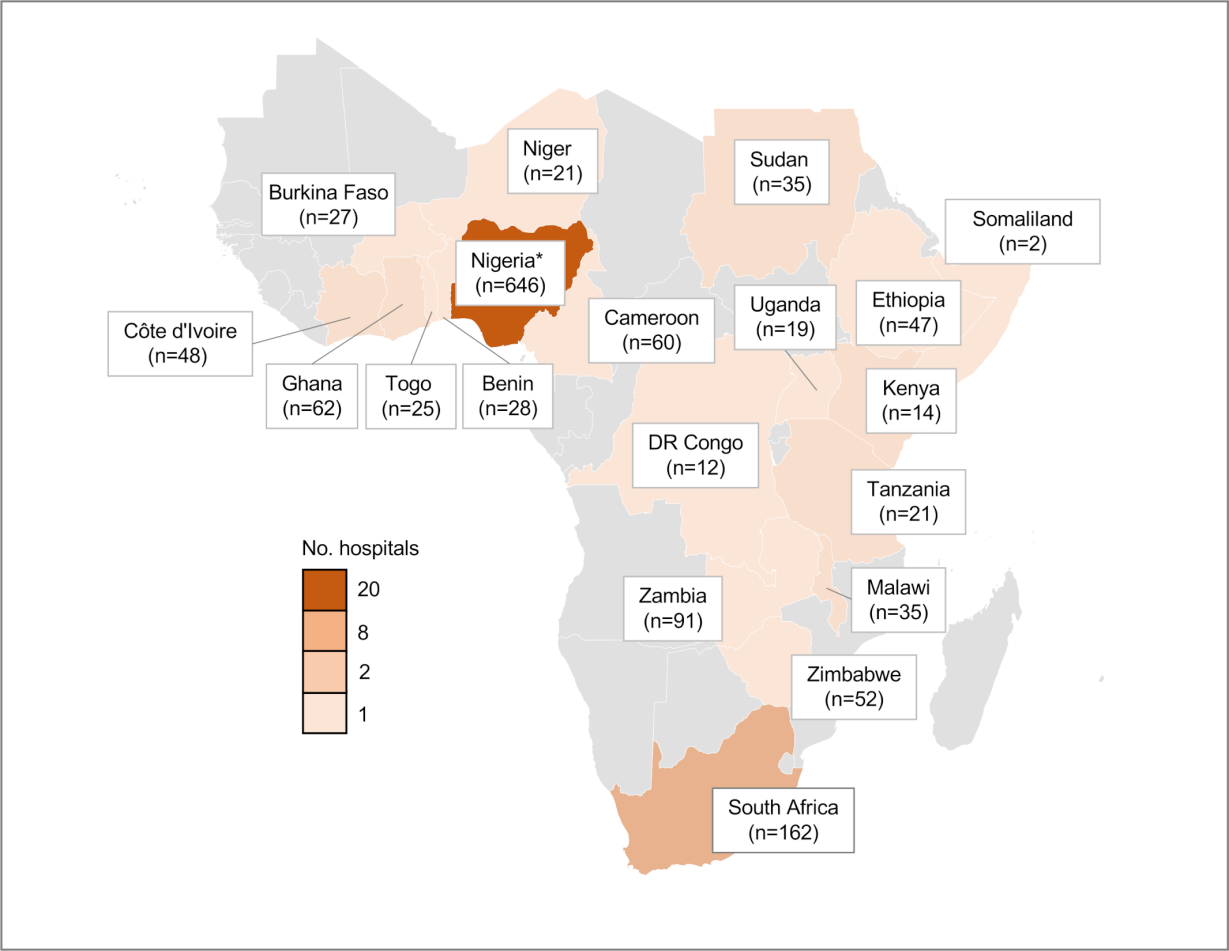
Countries in the study depicted by number of participating hospitals and patients (n). *The higher study population in Nigeria reflects the high population of the country (18% of the sub-Saharan Africa population) and the high number of paediatric surgeons (87 compared to a median of 4/country in sub-Saharan Africa).

Data were collected on 1407 children from 51 hospitals across 19 countries in SSA (figures 1 and 2): 111 patients with gastroschisis (7.9%), 188 with ARM (13.4%), 225 with intussusception (16.0%), 250 with appendicitis (17.8%) and 633 with inguinal hernia (45.0%). The data were collected over 75 one-month study periods. The median number of months of data collection per hospital was 1 month (range 1–4). The patient characteristics and perioperative management relevant to all conditions are presented in table 1. The condition-specific patient characteristics and management are reported in the online supplemental appendix tables 2a–e.

**Table 1:** Patient characteristics and perioperative care stratified by condition (generic variables only)

Variable	All, n=1407, n (%)	Gastroschisis, n=111, n (%)	ARM, n=188, n (%)	Intussusception, n=225, n (%)	Appendicitis, n=250, n (%)	Inguinal hernia, n=633, n (%)
Sex: male	1025 (72.9)	47 (42.3)	103 (54.8)	148 (65.8)	161 (64.4)	566 (89.4)
Gestational age at birth*	38, 37–40	37, 35–38	38, 37–39	39, 38–40	39, 38–40	38, 37–40
Weight (kg) on arrival*	10.0, 5.0–20.0	2.3, 2.0–2.7	3.3, 2.7–6.5	7.4, 6.4–8.6	30.0, 23.0–38.0	12.5, 7.9–18.0
Age*†	14, 4–72 (months)	1, 1–2 (days)	5, 2–92 (days)	7, 5–10 (months)	10, 8–12 (years)	25, 8–59 (months)
Condition onset to presentation (days)*	7, 2–127	1, 1–2	5, 2–92	3, 2–5	3, 2–5	120, 30–700
Patient’s home to hospital distance (km)*	20, 10–55	59, 18–182	47, 23–150	20, 10–45	17, 9–44	15, 8–35
Mode of transport to hospital:						
Ambulance or other health service transport	208 (14.9)	52 (47.3)	31 (16.5)	20 (9.0)	58 (23.7)	47 (7.5)
Patient’s own transportation	1168 (84.0)	50 (45.5)	153 (81.8)	203 (91.0)	187 (76.3)	575 (91.9)
Born in study hospital	15 (1.1)	8 (7.3)	3 (1.6)	NA	NA	4 (0.6)
ASA score:‡						
I-normal or II-mild systemic disease	1138 (82.9)	36 (36.4)	132 (76.3)	142 (64.8)	215 (86.0)	613 (97.0)
III-severe systemic disease	151(11.9)	20 (20.2)	27 (15.6)	56 (25.6)	34 (13.6)	14 (2.2)
IV/V-life threatening	84 (6.1)	43 (43.4)	14 (8.1)	21 (9.6)	1 (0.4)	5 (0.8)
NA, no surgical intervention	33	11	15	6	0	0
Surgical safety checklist used:‡						
No	631 (46.5)	60 (65.9)	77 (45.3)	125 (59.0)	111 (44.4)	258 (40.8)
Yes	725 (53.5)	31 (34.1)	93 (54.7)	87 (41.0)	139 (55.6)	375 (59.2)
NA, no surgical intervention	51	20	18	13	0	0
Anaesthetic:‡						
General anaesthetic	1101 (82.2)	36 (42.4)	152 (93.8)	172 (81.5)	239 (96.0)	502 (79.3)
No GA (local, regional, ketamine or none)	239 (17.8)	49 (57.7)	10 (6.2)	39 (18.5)	10 (4.0)	131 (20.7)
NA, no intervention	66	26	26	13	1	0
Anaesthetist:‡						
Anaesthetic doctor§	923 (70.5)	28 (35.9)	114 (70.4)	134 (70.9)	175 (70.3)	472 (74.7)
Anaesthetic nurse	294 (22.4)	9 (11.5)	42 (25.9)	44 (23.3)	64 (25.7)	135 (21.4)
Medical officer, surgeon or other	93 (7.1)	41 (52.6)	6 (3.7)	11 (5.8)	10 (4.0)	25 (4.0)
NA, no anaesthetic given	96	33	26	36	1	0
Blood transfusion:						
No, not required	1188 (84.6)	78 (70.3)	146 (78.9)	134 (59.6)	219 (87.6)	611 (96.5)
No, required but not available	21 (1.5)	8 (7.2)	3 (1.6)	2 (0.9)	2 (0.8)	6 (0.9)
Yes	195 (13.9)	25 (22.5)	36 (19.5)	89 (39.6)	29 (11.6)	16 (2.5)

Percentages have been rounded and may not total 100. Missing data: gestational age at birth n=199, weight n=21, age n=10, condition onset to presentation n=5, distance from home n=47, mode of transport n=16, ASA score n=1, anaesthetic n=1, anaesthetist n=1, blood transfusion n=3.

*Median, IQR.

†Age at presentation for patients with gastroschisis, ARM, intussusception and appendicitis. Age at the time of operation for patients with an inguinal hernia.

‡At primary intervention.

§Consultant or trainee.

ARM, anorectal malformation; ASA, American Society of Anesthesiologists; GA, general anaesthetic; NA, not applicable.

Few neonates with gastroschisis were diagnosed antenatally (n=5 (4.5%)) or born at the study hospital (n=8 (7.3%)). They frequently arrived without health service transportation (n=50 (45.5%)). Most presented on day 1 of life (IQR 1–2), but many were already septic (n=60 (54.5%)), hypovolaemic (n=64 (57.7%)) and hypothermic (n=84 (75.7%)). Similarly, there were high rates of sepsis at presentation for patients with ARM, intussusception and appendicitis (n=41 ((21.9%), n=102 (45.3%) and n=140 (56.0%), respectively) with median times to presentation of 3–5 days. ASA scores at primary intervention were high (3 or above), particularly in patients with gastroschisis, ARM and intussusception (n=63 (63.6%), n=41 (23.7%) and n=76 (35.2%), respectively) and a high proportion required blood transfusions (n=23 (29.7%), n=39 (21.1%) and n=91 (40.5%), respectively). Surgical pathology was frequently advanced: 31 (27.9%) patients with gastroschisis were complex, 118 (52.4%) patients with intussusception had a contraindication to non-operative management and 140 (57.1%) patients with appendicitis had perforated. In contrast, patients with an inguinal hernia had lower ASA scores (3% ASA 3–5). Of the 633 patients with an inguinal hernia, 59 (9.3%) were complicated (57 (9.0%) incarcerated, obstructed or strangulated and 2 (0.3%) fistulated).

Deficiencies in primary resuscitation were identified: 19 (22.6%) hypothermic neonates with gastroschisis were not warmed, while 20 (11.4%) patients with intussusception and 13 (7.7%) patients with appendicitis were not given intravenous fluids for sepsis or hypovolaemia prior to surgical intervention. Ventilation was not available when required for 36 (32.4%) patients with gastroschisis and 60 (32.0%) with ARM. 85 (76.6%) patients with gastroschisis had no or insufficient parenteral nutrition. Some patients with gastroschisis, ARM and intussusception were palliated without a surgical intervention (n=20 (18.2%), n=7 (3.8%) and n=7 (3.1%), respectively). Minimally invasive or non-operative techniques were used infrequently: 25 (10.0%) of patients with appendicitis were managed laparoscopically and 58 (25.8%) patients with intussusception had an air enema or hydroenema reduction (successful in 40 (69.0%)). Of patients undergoing laparotomy for intussusception, 63 (35.6%) underwent manual reduction only. Management of gastroschisis and ARM varied widely.

### Hospital characteristics

Forty-eight (94%) participating hospitals provided survey data regarding their availability of paediatric surgical resources (table 2).

**Table 2:** Hospital resources available for paediatric surgery in sub-Saharan Africa (n=48 hospitals)

Hospital resource	Availability, % (n)
Personnel*	
Number of paediatric surgeons undertaking general paediatric surgery/hospital:	
0	8.7 (4)
1–3	65.2 (30)
4 or more	26.1 (12)
Number of paediatric surgeons undertaking neonatal surgery/hospital:	
0	10.9 (5)
1–3	71.7 (33)
4 or more	17.4 (8)
Number of general surgeons undertaking general paediatric surgery/hospital	
0	47.8 (22)
1–3	32.6 (15)
4 or more	19.6 (9)
Number of general surgeons undertaking neonatal surgery/hospital:	
0	67.4 (31)
1–3	21.7 (10)
4 or more	10.9 (5)
Number of medical officers undertaking general paediatric surgery/hospital:	
0	78.3 (36)
1–3	15.2 (7)
4 or more	6.5 (3)
Number of medical officers undertaking neonatal surgery/hospital:	
0	93.5 (43)
1–3	2.2 (1)
2 or more	4.3 (2)

	Always available, % (n)	Sometimes/never available, % (n)
**Infrastructure**		
Running water	93.8 (45)	6.2 (3)
Electricity	52.1 (25)	47.9 (23)
Electricity back-up generator	64.6 (31)	35.4 (17)
Laboratory for biochemistry	66.7 (32)	33.3 (16)
Laboratory for haematology	75.0 (36)	25.0 (12)
Blood bank	83.3 (40)	16.7 (8)
Functioning ultrasound machine	79.2 (38)	20.8 (10)
Fluoroscopy	27.1 (13)	72.9 (35)
Paediatric ventilation outside the operating room	31.3 (15)	68.7 (33)
Neonatal ventilation outside the operating room	31.3 (15)	68.7 (33)
PICU for children requiring surgical care	29.2 (14)	70.8 (34)
NICU for neonates requiring surgical care	35.4 (17)	64.6 (31)
Parenteral nutrition	29.2 (14)	70.8 (34)
Surgical safety checklist in the operating room	43.8 (21)	56.3 (27)
Sterile gloves and gown	89.6 (43)	10.4 (5)
Autoclave for sterilising surgical equipment	89.6 (43)	10.4 (5)
Peña simulator for anorectal reconstruction	33.3 (16)	66.7 (32)
**Procedures**		
Bianchi cotside closure of gastroschisis	27.1 (13)	72.9 (35)
Performed silo application, reduction and closure	22.9 (11)	77.1 (37)
Surgical silo application, reduction and closure	31.3 (15)	68.8 (33)
Primary closure of gastroschisis in the operating room	45.8 (22)	54.2 (26)
Sigmoid colostomy	83.3 (40)	16.7 (8)
Posterior sagittal anorectoplasty	85.4 (41)	14.6 (7)
Open appendectomy	93.8 (45)	6.3 (3)
Laparoscopic appendectomy	18.8 (9)	81.2 (9)
Ultrasound guided drainage of intra-abdominal collection	35.4 (17)	64.6 (31)
CT guided drainage of intra-abdominal collection	16.7 (8)	83.3 (40)
Personnel to diagnose intussusception on ultrasound	75.0 (36)	25.0 (12)
Air-enema reduction of intussusception	20.8 (10)	79.2 (38)
Hydro-enema reduction of intussusception	29.2 (14)	70.8 (34)
Laparotomy for intussusception	87.5 (42)	12.5 (6)
Open herniotomy	97.9 (47)	2.1 (1)
Laparoscopic herniotomy	12.5 (6)	87.5 (42)
Paediatric central line	33.3 (16)	66.7 (32)
Neonatal central line	29.2 (14)	70.8 (34)
Umbilical vein catheterisation	50.0 (24)	50.0 (24)
**Anaesthesia and resuscitation**		
Paediatric bag, valve and mask	85.4 (41)	14.6 (7)
Bottled oxygen	77.1 (37)	22.9 (11)
Piped oxygen	56.3 (27)	43.7 (21)
Oxygen saturation monitor	75.0 (36)	25.0 (12)
Apnoea monitor	39.6 (19)	60.4 (29)
Multiparameter intraoperative monitoring	79.2 (38)	20.8 (10)
Anaesthetic machine for children	66.7 (32)	33.3 (16)
Anaesthetic machine for neonates	52.1 (25)	47.9 (23)
Ketamine anaesthesia for children	60.4 (29)	39.6 (19)
Ketamine anaesthesia for neonates	43.8 (21)	56.2 (27)
Spinal/caudal anaesthesia for children	31.3 (15)	68.7 (33)
Spinal/caudal anaesthesia for neonates	29.2 (14)	70.8 (34)
Anaesthetic doctor to undertake paediatric anaesthesia†	70.8 (34)	29.2 (14)
Anaesthetic doctor to undertake neonatal anaesthesia†	35.4 (17)	64.6 (31)
Anaesthetic nurse to undertake paediatric anaesthesia†	56.2 (27)	43.8 (21)
Anaesthetic nurse to undertake neonatal anaesthesia†	43.8 (21)	56.2 (27)

46/48 hospitals provided data on personnel. 48/48 hospitals provided data for all other categories.

*Includes consultant surgeons only.

†Includes all grades/training levels.

NICU, neonatal intensive care unit; PICU, paediatric intensive care unit.

The majority of hospitals had between one to three consultant paediatric surgeons for paediatric and neonatal surgery (n=30 (65%) and n=33 (72%) hospitals, respectively). Paediatric and neonatal intensive care were reliably available at just 14 (29%) and 17 (35%) hospitals, respectively. Only 14 (29%) hospitals had reliable access to parenteral nutrition. Central venous access was consistently available in only 16 (33%) hospitals for children and 14 (29%) hospitals for neonates. Reliable availability of specialist equipment was also limited: preformed silo use for gastroschisis (n=11 (23%) hospitals), air or hydroenema reduction for intussusception (n=14 (29%) and n=10 (21%) hospitals, respectively) and laparoscopic appendicectomy (n=9 (19%) hospitals). An anaesthetic doctor (including all levels of training) was available to undertake paediatric anaesthesia at 34 (71%) hospitals and neonatal anaesthesia at 17 (35%) hospitals.

An additional 23 hospitals (online supplemental appendix figure 1) provided survey data without patient data. Hospital characteristics were compared between these 23 hospitals and the 48 hospitals in the study using χ^2^ statistics and no differences were found at p<0.10.

### Outcomes

**Figure 3:**
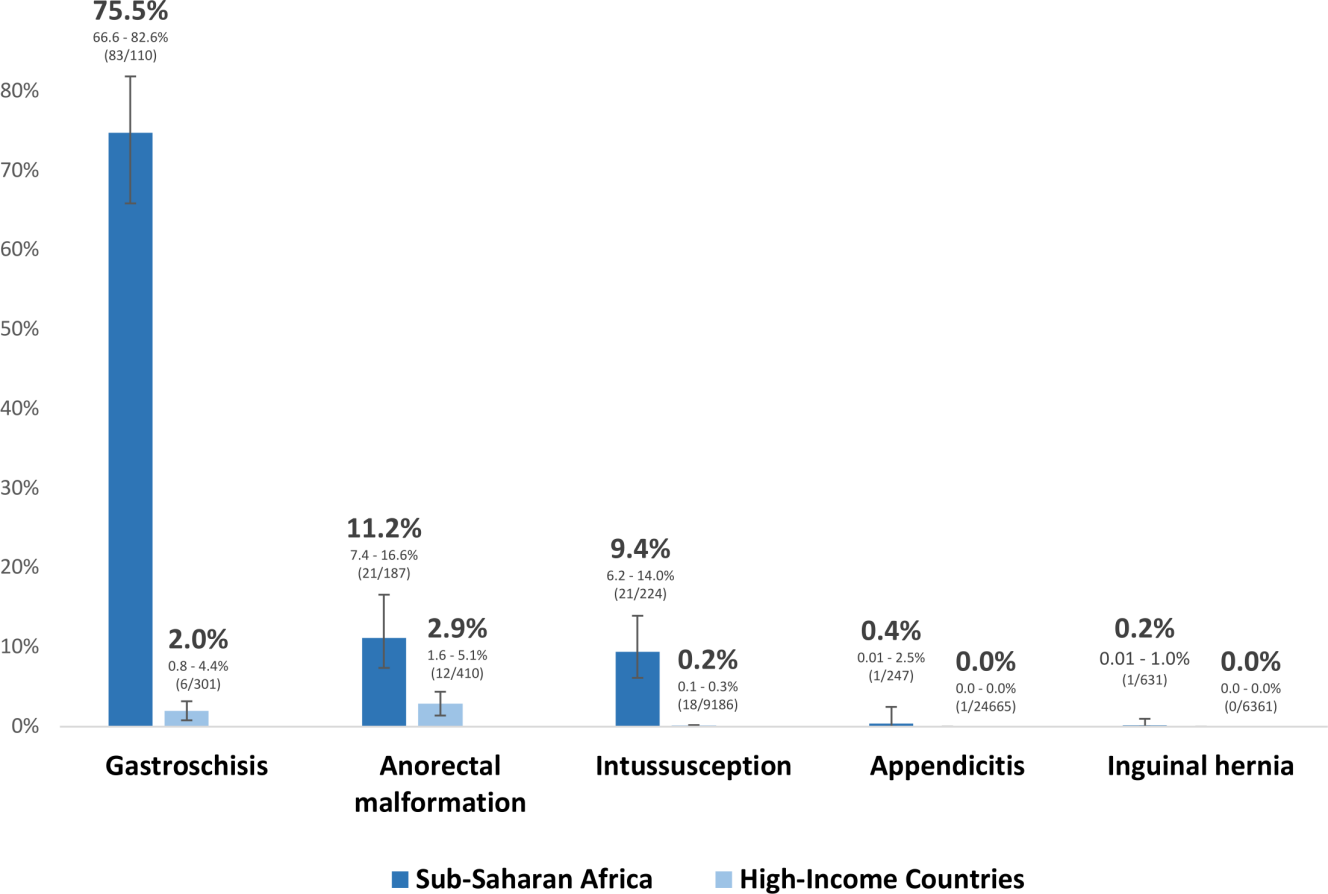
A comparison between the mortality in sub-Saharan Africa and published benchmark mortality in high-income countries, with 95% CIs.^10 15 16 20 25^

There was a significant disparity in mortality between SSA and HICs for all paediatric surgical conditions: gastroschisis (75.5% vs 2.0%, p<0.0001), ARM (11.2% vs 2.9%, p<0.0001), intussusception (9.4% vs 0.2%, p<0.0001), appendicitis (0.4% vs 0.004%, p=0.02) and inguinal hernia (0.2% vs 0.0%, p=0.008), respectively (figure 3).^10 15 16 20 25^ Mortality was highest among neonates (112/267, 41.9%), followed by infants (20/403, 5.0%) and children (7/720, 1.0%) (online supplemental appendix table 3).

Secondary outcomes are detailed in table 3. Eleven patients with ARM and one patient with an inguinal hernia died following discharge within 30-days of primary intervention. Hence, the 30-day post-primary intervention mortality for these conditions in SSA was 17.0% and 0.3%, respectively. Condition specific complications are detailed in online supplemental appendix tables 2a to 2e and patient follow-up in online supplemental appendix table 4.

**Table 3:** Secondary outcomes

	Surgical site infection, % (number of cases/sample size)	Wound dehiscence, % (number of cases/sample size)	Unplanned reintervention, % (number of cases/sample size)	30-day post-intervention mortality % (number of cases/sample size)*	Duration of hospital stay among patients who survived to discharge (days)†	Duration of hospital stay among patients who died in hospital (days)†
Gastroschisis	21.1 (20/95)	3.2 (3/94)	16.0 (15/94)	74.8 (83/111)	16.5, 7–24	4, 2–7
Anorectal malformation	23.1 (37/160)	9.4 (15/160)	11.2 (19/170)	17.0 (32/188)	7, 5–14	3, 2–5
Intussusception	25.4 (45/177)	7.3 (13/177)	11.6 (26/224)	9.3 (21/225)	7, 5–11	3, 2–5
Appendicitis	18.5 (45/243)	4.9 (12/243)	9.9 (24/243)	0.4 (1/250)	5, 4–8	3
Inguinal hernia	4.2 (26/625)	1.0 (6/625)	1.3 (8/624)	0.3 (2/633)	1, 1–3	1

*Study population used as denominator; 30-day post intervention mortality status unknown for 16.4% patients (online supplemental appendix table 4).

†Median, IQR. One patient died in each of the appendicitis and inguinal hernia categories, hence IQR not available.

On multivariable analysis of patients with gastroschisis, ARM and intussusception combined (generic patient variables only), the following were significant predictors of mortality (OR (95% CI), p value): ARM vs gastroschisis (0.07 (0.02 to 0.18), <0.001), intussusception versus gastroschisis (0.03 (0.01 to 0.10), p<0.001), ASA score 3–5 vs 1–2 at primary intervention (3.91 (1.74 to 8.79), p=0.001), receiving a blood transfusion (3.72 (1.59 to 8.68), p=0.002) and not receiving anaesthesia due to no surgical intervention (10.48 (2.07 to 52.97), p=0.004) (online supplemental appendix table 5). In the second multivariable model incorporating hospital-level factors, the same patient-level factors remained significant; no hospital factors were significant.

### Exploratory analysis

On univariable analysis of patients with gastroschisis, ARM and intussusception, individually, the generic variables significantly associated with mortality reflect those identified in the multivariable model of all three conditions combined (online supplemental appendix tables 6-8). Condition-specific variables associated with a higher mortality were (OR (95% CI), p value): complex gastroschisis (4.39 (1.12 to 17.20), p=0.034) and hypovolaemia on arrival (7.84 (1.96 to 31.34), p=0.004) for patients with gastroschisis; sepsis on arrival (8.55 (2.89 to 25.34), p<0.001) and electrolyte disturbance (29.94 (6.31 to 142.05), p<0.001) and sepsis (22.20 (5.05 to 97.68), p<0.001) within 30 days of surgery, for patients with ARM; and sepsis on arrival (14.59 (3.10 to 68.70), p=0.001) and shock (8.98 (2.21 to 36.50), p=0.002) for patients with intussusception. Not having associated anomalies (0.24 (0.08 to 0.73), p=0.012) and not requiring ventilation (0.24 (0.07 to 0.82), p=0.022) were associated with a lower mortality for patients with ARM. Higher procedure score was associated with a lower mortality for gastroschisis (0.16 (0.34 to 0.84), p=0.031); no hospital-level variables were significantly associated with mortality for ARM and intussusception.

### Data validation

Validation of the patient-level data showed very good agreement (observed agreement 94%; median kappa statistic 0.96) between the main study and validation datasets (online supplemental appendix table 9). Validating investigators only identified one patient (with an inguinal hernia) who was eligible for study inclusion, but not included. The validation survey identified the following variables as higher risk for inaccuracy: gestational age, distance from home to hospital, and time from arrival to intervention (online supplemental appendix table 10). Validation of the hospital data showed fair agreement between investigators independently completing the survey at each hospital (observed agreement 75%; median interclass correlation coefficient 0.58) (online supplemental appendix table 11).

## Discussion

This multicentre, multinational prospective cohort study highlights significantly higher mortality rates for common paediatric surgical conditions in SSA compared with published benchmark mortality rates in HICs, particularly for neonates. The disparity in mortality was greatest in patients with gastroschisis (75.5% vs 2.0%), ARM (11.2% vs 2.9%) and intussusception (9.3% vs 0.2%), respectively. Correspondingly, overall mortality was highest among neonates (41.9%) compared with infants (5.0%) and children (1.0%). These results are consistent with the GlobalSurg Study on emergency abdominal surgery outcomes, which also reported much higher mortality rates in neonates (18%–24%) compared with infants (9%) and children (1%–4%) in low-income and middle-income countries (LMICs), globally.^29^ Our findings align with a systematic review of neonatal surgical outcomes in SSA, which reported an overall mortality of 31.8%, and over 50% mortality for emergency neonatal surgery involving gastrointestinal congenital anomalies such as gastroschisis.^8^

Congenital anomalies have recently risen to become the fifth-leading cause of death in children under 5 globally (half a million annual deaths) and 11th leading cause of years of life lost for the global population.^3 30^ A recent study from Ghana highlighted that congenital anomalies accounted for 87% of neonatal surgical cases and 96% of deaths; two-thirds of the congenital anomaly deaths were in conditions involving the gastrointestinal tract.^31^ It is estimated that two-thirds of the deaths and disability from congenital anomalies can be prevented through surgical care.^32^ However, improving neonatal surgical care has received little global focus or action to date.^33^ Deaths from congenital anomalies fell very little from 2005 to 2015 (-3.2%) compared with the other leading causes of under 5 mortality (−25.9% preterm birth, −16.1% neonatal encephalopathy, −36.9% lower respiratory infections, −34.3% diarrhoeal diseases, −42.8% malaria).^3^ Sustainable Development Goal (SDG) 3.2 aims to ‘end preventable deaths in neonates and children under 5 by 2030’.^34^ The high mortality among neonates with congenital anomalies in this study, and in the systematic review of neonatal surgical outcomes in SSA, highlight the urgent need to target improvements in neonatal surgical care for this to be achieved.

Higher ASA score at primary intervention, needing/receiving a blood transfusion and paediatric surgical condition, were significantly associated with mortality on multivariable analysis of generic variables related to patients with gastroschisis, ARM and intussusception. Exploratory analysis of the condition-specific variables for the individual conditions, although only univariable and hence less robust, provides some further insights; higher mortality was significantly associated with having complex gastroschisis and hypovolaemia on arrival for patients with gastroschisis, and sepsis on arrival for patients with ARM and intussusception. Indeed, the high proportion of patients presenting with sepsis, hypovolaemia and other indicators of advanced disease severity, account for the large proportion of patients with high ASA scores at primary intervention. Some deficiencies were also identified in preintervention resuscitation which may also impact on the ASA score. The surgical condition determines how quickly clinical deterioration can progress without appropriate care. Patients with gastroschisis deteriorate most rapidly due to the large surface area of exposed bowel resulting in hypothermia, hypovolaemia and sepsis, within hours if plastic bowel coverage and resuscitative measures are not instigated at birth.^35^ Hence, interventions to improve outcomes must focus on improving resuscitation and timely referral/transportation at the district level, and preintervention resuscitation and perioperative care at paediatric surgery centres. This aligns with recommendations by Kruk *et al* that to achieve improved outcomes in the SDG era, enhancing access to services must be accompanied by improved quality of care within facilities.^36–38^ Studies from individual paediatric surgical centres in SSA have reported similar findings and conclusions.^39^

The hospital survey highlighted numerous deficiencies in resources required to provide high-quality paediatric surgical care; less than a third of hospitals had ventilation, parenteral nutrition and central intravenous access facilities for neonates. Our findings are consistent with those of the PediPIPES hospital survey undertaken in West Africa which reported that over half of the hospitals had less than three paediatric surgeons, half had no neonatal or paediatric intensive care, life-saving equipment such as apnoea monitors were deficient, and minimally invasive interventions such as non-operative reduction of intussusception were frequently unavailable.^40^ Interestingly, we found that hospital-level factors were not significantly associated with mortality on multivariable analysis. This may be related to insufficient patient numbers, but also may mirror other studies highlighting that resources alone do not determine outcomes; it is the actions undertaken by an effective workforce using the available resources, that result in improved care.^36 41^ Indeed, higher procedure availability, incorporating both skilled healthcare personnel and resources, was associated with lower mortality on univariable analysis for patients with gastroschisis.

The Global Initiative for Children’s Surgery (GICS) provides a framework for improving children’s surgical care and outcomes in LMICs within four main domains: infrastructure, service delivery, training and research.^42^ GICS has produced the Optimal Resources for Children’s Surgery guidelines detailing what is required within each domain at every level of healthcare.^43^ This has recently been used alongside a paediatric modified WHO assessment tool to help integrate children’s surgical care into the Nigerian National Surgical, Obstetric and Anaesthetic Plan (NSOAP).^44 45^ This can be used as a template for other SSA countries and LMICs globally, to include children’s surgical care within National Health Plans. Government engagement and investment will be essential to ensure implementation of NSOAPs and ultimately improved surgical outcomes.^46^ This study highlights the importance of ensuring neonatal surgical care is a key focus of these plans as it is where a large proportion of the mortality burden lies.

International and local partnerships can help support the government with specific NSOAP goals. For example, the KidsOR Charity is investing in building children’s operating facilities, and training paediatric surgeons and anaesthetists, across SSA.^47^ The hub and spoke model used in India whereby multidisciplinary teams (MDTs) from paediatric surgical centres provide outreach teaching to district hospital teams and create referral networks, is an excellent example of how local partnerships can help to optimise district level care.^48^ However, to enable fast and safe transfer when needed, this must be coupled with investment into the transport system. In response to the results of this study, a multicentre, multinational interventional study has been funded aimed at reducing mortality from gastroschisis in SSA.^49 50^ In addition to outreach teaching and establishment of referral networks, it incorporates evidence-based protocolised care at the paediatric surgical centres with a focus on resuscitation at presentation, perioperative care and MDT collaboration; a model that could benefit neonatal and paediatric surgical conditions more broadly.^51^ Locally sourced parenteral nutrition and low-cost preformed silos were included, highlighting the importance of investing in vital resources alongside workforce interventions.

Further partnerships are required with obstetric teams to optimise antenatal diagnosis and delivery of neonates with congenital anomalies such as gastroschisis, at paediatric surgery centres. Just 4.5% of neonates with gastroschisis were antenatally diagnosed in this study and 7.3% born at a tertiary centre; these interventions, which are standard practice in HICs, would avoid clinical deterioration before reaching paediatric surgical care. Partnerships with existing child health programmes, such as the WHO Every Newborn Action Plan, are required to optimise early recognition, resuscitation and safe transfer of surgical neonates and children at the community and district level.^46 52 53^ In addition, there is a need for further research into the management and outcomes of a wider selection of congenital anomalies requiring surgical care in SSA, particularly congenital anomalies involving the gastrointestinal tract which are commonly fatal without timely access to quality neonatal surgical care following birth. Following on from this study, the PaedSurg Africa Research Collaboration has been expanded to form the Global PaedSurg Research Collaboration with the first study focused on the management and outcomes of a wider selection of common gastrointestinal congenital anomalies in low, middle and HICs, globally.^54^

### Limitations

Although this study provides some of the most comprehensive, high-quality, validated paediatric surgical outcome data from SSA, there are limitations. The study covered just 19 of the 48 countries in SSA. However, the sample was reasonably representative containing 9 low-income countries (LICs), 9 lower-middle income countries and 1 upper-middle income country (UMIC) compared with 14 LICs, 9 lower-middle income countries, 4 UMICs and 2 HICs that were not included. All geographical regions (West, East, Central and South Africa) were represented. Nigeria accounted for 644 (45.8%) of the study patients. This reflects the high population in Nigeria (18% of the SSA population) and high number of paediatric surgeons (87 compared with a median of 4/country across SSA).^5^ The overall 30-day post intervention mortality was 6.2% (40/644) in Nigeria and 13.0% (99/763) among the other 18 countries. Hence, mortality rates for the paediatric surgical conditions across all SSA countries could be slightly higher than reported. Although some countries in chronic conflict, such as the Democratic Republic of the Congo and Sudan were included, the study was not specifically designed to address the unique challenges faced in these regions, which may have an impact on mortality.

There are a number of additional reasons why the mortality and morbidity may be underestimated. Data collection was at an institutional level rather than population level and some patients may have died before reaching the study centres.^55 56^ In contrast, the hospital data from HICs likely reflects the population mortality due to better access and higher-quality antenatal services, district-level care and transportation. Indeed the HIC comparator mortality rates used may be an overestimate of the current mortality in these regions due to further reductions over time, particularly for the ARM data, which was published in 2008.^15^ 30-day post intervention mortality status was not known for 16.4% patients and hence some deaths following discharge could have been missed, as could complications. A convenience sample of self-selected paediatric surgery centres was used, which could represent the more academic hospitals with better outcomes. Participating hospitals with greater human resource capacity could have been more likely to contribute a greater number of months of data to the study. Bias was minimised by adjusting both the univariable and multivariable analyses for clustering at a hospital level. Patients with appendicitis and inguinal hernia could also be managed at district hospitals where paediatric surgical and anaesthetic expertise are likely lower and less resourced.

For feasibility reasons, the study included just five common paediatric surgical conditions rather than all paediatric surgical cases. Inclusion of gastroschisis in the selection of study conditions, with such a high mortality, may have elevated the overall neonatal mortality in comparison to the broader range of neonatal surgical conditions in SSA. However, our results are similar to the systematic review of all neonatal surgical conditions in SSA.^8^ In contrast, the true mortality from the broader range of paediatric surgical conditions among children over 1 year of age in SSA may be higher. This is because acute abdomen in children in HICs is mostly related to appendicitis, but in SSA typhoid perforation is also prevalent, with reported mortality of 14.7%.^57^ To optimise feasibility, data collection was limited to information that would be known to the team caring for the study patients without the need for additional measurements. Hence, variables such as weight-for-length/height z-score to classify nutritional status were not included, but could have an impact on outcomes.

Finally, HIC benchmark data were used as the comparator. The HIC studies selected had reasonable comparability in terms of duration of follow-up, patient population and study setting, however it would be more accurate to compare observed outcomes in SSA directly with HIC outcomes collected simultaneously within the same study. This has been incorporated into our follow-on study, Global PaedSurg, where outcomes from gastrointestinal congenital anomalies will be collected globally.^54^ The latter will also facilitate collection of larger sample sizes to provide greater insights into factors affecting mortality that can be modified to improve outcomes.

## Conclusions

This study highlights unacceptably high mortality from common paediatric surgical conditions in SSA compared with HICs, particularly for neonates. It provides data to support the assertion that SDG 3.2 to end preventable deaths in neonates and children under 5 by 2030 will not be achieved without global focus, considerable investment and action to improve paediatric surgical care in SSA.

## Data Availability

Once published, the full anonymous, de-identified patient and hospital datasets will be made publicly available via the Centre for Open Science website: https://osf.io/72pkj/.
