# Supplementary material for: Paediatric surgical outcomes in sub-Saharan Africa: a multicentre, international, prospective cohort study
Source: BMJ Glob Health. 2021 Sep 2;6(9):e004406. doi: 10.1136/bmjgh-2020-004406 (PMC8413881; doi:10.1136/bmjgh-2020-004406)
Supplement: Supplementary data [file bmjgh-2020-004406supp001.pdf]

Paediatric Surgical Outcomes in Sub-Saharan Africa: A Multicentre, International, Prospective Cohort Study

PaedSurg Africa Research Collaboration

SUPPLEMENTAL APPENDIX

Appendix Methods 1. Patient Data Collection Form
Appendix Methods 2. Hospital Survey
Appendix Methods 3. Power calculation
Appendix Figure 1. Additional hospitals providing survey data without patient data
Appendix Table 1. High-income country benchmark data used as a comparator to observed SSA data
Appendix Tables 2a-2e. Condition specific patient characteristics, management and outcomes
Appendix Table 3. Mortality stratified by age
Appendix Table 4. Patient follow-up
Appendix Table 5. Univariable and multivariable analysis of patient-level and hospital-level factors associated with mortality for patients with gastroschisis, anorectal malformation and intussusception combined (generic patient variables only)
Appendix Table 6. Univariable analysis of patient-level and hospital-level factors associated with mortality for patients with gastroschisis (generic and condition specific variables)
Appendix Table 7. Univariable analysis of patient-level and hospital-level factors associated with mortality for patients with ARM (generic and condition specific variables)
Appendix Table 8. Univariable analysis of patient-level and hospital-level factors associated with mortality for patient with intussusception (generic and condition specific variables)
Appendix Table 9. Summary of the agreement between the main study and validation datasets
Appendix Table 10. Validation survey undertaken by local investigators at validating centers
Appendix Table 11. Validation of the hospital data

Appendix Methods 1. Patient Data Collection Form

Generic variables for all conditions

REDCap Record ID:

Demographics

- Gestational age at birth:

The number of weeks from the first day of the woman's last menstrual cycle until birth.

- Sex: Male/ Female
- Weight:

In kilograms (kg) on the day of presentation. Answer to one decimal place.

- Time from onset of the condition to this presentation at your hospital (in days):

For example: Number of days from birth until presentation for gastroschisis and anorectal malformation. Time from onset of symptoms to presentation for appendicitis and intussusception. Time from first noticing the hernia to the first day of this admission at your hospital for patients with an inguinal hernia. Please include the first day and day of presentation in your calculation, for example, a patient presenting with appendicitis on 5th October 2016 who has had abdominal pain since 1st October 2016, please insert 5 days.

- Distance from the patient's home to your hospital:

In kilometres (km). Please round to the nearest kilometer.

- Mode of transport to your hospital:

Ambulance/ Other transport method provided by the health service/ Patient's own transport/ Born within your hospital

Peri-operative Factors

- American Society of Anesthesiologists (ASA) score at the time of primary surgery or intervention:

1. healthy person/ 2. mild systemic disease/ 3. severe systemic disease/ 4. severe systemic disease that is a constant threat to life/ 5. a moribund patient who is not expected to survive without the operation/ Not applicable - intervention or surgery not undertaken

- Was a Surgical Safety Checklist used at the time of primary surgery or intervention?
  Yes/ No/ Surgery or intervention not undertaken
- What type of anaesthesia was used for the primary intervention?

General anaesthesia with endotracheal tube/ General anaesthesia with laryngeal airway/ Ketamine anaesthesia/ Spinal or caudal anaesthesia/ Local anaesthesia/ No anaesthesia, just analgesia/ No anaesthesia, no analgesia/ Not applicable: no surgery or intervention undertaken.

For gastroschisis please enter the anaesthesia used at the time of defect closure. For intussusception please enter the anaesthesia used for the primary intervention whether it was an air- or hydro-enema or primary surgery.

- Who undertook the anaesthetic for the primary intervention?

Anaesthetic doctor/ Anaesthetic nurse/ Medical officer/ Other/ No anaesthetic undertaken

If other, please specify:

- Did the patient receive a blood transfusion during their primary admission?

No: not required/ No: it was required but not available/ Yes: not cross-matched/ Yes: cross-matched.

Indications for blood transfusion: a haemoglobin level less than 80g/L associated with symptoms including tachypnoea, shortness of breath and tachycardia.

Outcomes

- Did the patient survive to discharge? Yes/ No

If the patient was still in your hospital and alive at 30 days after primary admission, please select yes.

- If you were able to follow-up the patient, were they alive at 30 days following primary surgery/ intervention? Yes/ No/ Not followed up after discharge/ Not followed up to 30 days post-intervention

Follow-up includes either in person, via telephone or other means of reliable communication.

- Duration of hospital stay (days):

Please include the day of admission and the day of discharge in your calculation. For example, if a patient presented with appendicitis on 1st October and was discharged on the 5th October, their duration of hospital stay would be 5 days. If the patient died, please record the number of days from admission to death. Only include the duration of the primary admission; not subsequent admissions if the patient re-presented. If the operation was undertaken as a daycase and the patient was discharged the same day, please enter 1.

- Did the patient have a surgical site infection? Yes/ No

Surgical site infection criteria include at least one of the following: 1) purulent drainage from the superficial or deep (fascia or muscle) incision but not from within the organ/space component of the surgical site, 2) at least one of: pain or tenderness; localised swelling; redness; heat; fever; AND the incision is opened deliberately or spontaneously dehisces, 3) there is an abscess within the wound (clinically or radiologically detected).

- Did the patient have a full thickness wound dehiscence? Yes/ No

This is defined as all wound layers opening.

- Did the patient require a further intervention within 30 days of primary surgery?

No/ Yes - percutaneous intervention/ Yes - surgical intervention

. *BMJ Global Health* 2021; 6:e004406. doi: 10.1136/bmjgh-2020-004406

- If the patient had a complication, when was it diagnosed?

During the primary admission/ As an emergency re-attender/ At routine follow-up as an out-patient/ Not applicable, no complications

- Did the patient require re-admission to hospital? Yes/ No
- If re-admission was required, what was the duration of their subsequent hospital stay (in days)?

Please include the day of admission and the day of discharge in your calculation. For example, if a patient re-presented on 1st November and was discharged on the 5th November, their duration of hospital stay would be 5 days. If the patient died, please record the number of days from admission to death.

- Was the patient followed up at 30 days post primary surgery or intervention to assess for complications?

No: data is based on in-patient observations only/ No: follow-up was done, but prior to 30 days/ Yes: reviewed in person/ Yes: via telephone consultation/ Yes: via other means/ Yes: still an in-patient at 30 days

- What study condition does this patient have?

Gastroschisis/ Anorectal malformation/ Appendicitis/ Intussusception/ Inguinal hernia

---

Condition specific variables

Gastroschisis

- Type of gastroschisis:

Simple/ Complex: associated with atresia/ Complex: associated with necrosis/ Complex: associated with perforation

- Was the gastroschisis diagnosed antenatally? Yes/ No
- Was the neonate septic on arrival to your unit? Yes/ No

Sepsis is SIRS (Systemic Inflammatory Response Syndrome) with a suspected or confirmed bacterial, viral, or fungal cause. SIRS is a response to a stimulus, which results in two or more of the following: temperature > 38.5°C or < 36°C, tachycardia (> two standard deviations above normal), or bradycardia in children < 1 year old (< 10th centile for age), tachypnoea (> two standard deviations above normal), leukocyte count > 12,000 cells/mm3, < 4,000 cells/mm3, hyperglycemia, altered mental status, hyperlactemia, increased capillary refill time >2 seconds.

- Did the neonate receive broad spectrum antibiotics within an hour of arrival to your unit?

Yes/ No: not given within 1 hour, but they were given on the first day of admission/ No: not given on the first day of admission/ Broad spectrum antibiotics cover gram negative, gram positive and anaerobic bacteria.

- Were broad spectrum antibiotics continued for at least 48 hours after abdominal wall closure? Yes/ No

Or until death if the neonate did not survive until abdominal wall closure.

- If the neonate was hypothermic on arrival to your unit, were they warmed with an incubator or radiant heater?

Yes/ No/ Not applicable - not hypothermic

Hypothermia is defined as < 36.5°C core temperature.

- Was the neonate hypovolaemic on arrival to your unit? Yes/ No

Hypovolaemia criteria include: prolonged capillary refill time >2 seconds, and/ or tachycardia (> two standard deviations above normal for age), mottled skin, reduced urine output (< 1ml/kg/hr), cyanosis, impaired consciousness, hypotension (mean arterial pressure < 5th percentile for age).

- Did the neonate receive an intravenous fluid bolus within an hour of arrival on your unit?

Yes/ No: not within 1 hour, but it was given on the first day of admission/ No: not given on the first day of admission. Please select 'yes' only if 10ml/kg or more of intravenous fluid was administered.

- What intravenous access was achieved for the neonate within the first day of arrival on your unit?

None/ Peripheral cannula/ Umbilical vein catheter/ Central line (including all types other than via the umbilical vein). Please tick all that apply.

- Time from admission to primary intervention (in hours):

Please round to the nearest hour. If a preformed silo was used, please enter the time from admission to the first application of the performed silo.

- Method of gastroschisis closure:

Not attempted (palliative care)/ Primary closure at the bedside (Bianchi technique)/ Preformed silo application, reduction and closure at the bedside/ Preformed silo, reduction and closure in the operating room/ Surgical silo, reduction and closure in the operating room/ Primary closure in the operating room/ Other.
If other, please specify:

- On what day following admission was abdominal wall closure achieved?

Please include the first day of admission and the day of closure in the calculation. For example, for a neonate admitted with gastroschisis on 2nd October and had the gastroschisis reduced and closed on 4th October, please insert 3 days.

- If ventilatory support was required at any point during the care of this neonate, was it available?

Yes/ No/ Not applicable - not required

- Did this neonate receive parenteral nutrition?

Yes, until full enteral intake was established/ Yes, but for less time than was required/ No

. BMJ Global Health 2021; 6:e004406. doi: 10.1136/bmjgh-2020-004406

- Did the neonate have any of these complications within 30 days following primary intervention?

Ischaemic bowel/ Central line sepsis/ Abdominal compartment syndrome (defined as respiratory insufficiency secondary to compromised tidal volumes, decreased urine output caused by falling renal perfusion or any other organ dysfunction caused by increased intra-abdominal pressure)

If the patient had signs of abdominal compartment syndrome, was the abdomen re-opened?

Anorectal malformation

- Type of anorectal malformation:

Perineal or vestibular - able to pass stool spontaneously/ Higher malformation

- Does the neonate have an addition anomaly?

None/ Vertebral, Cardiac/ Oesophageal atresia/ Tracheoesophageal fistula/ Renal/ Limb/ Other

- Was the baby septic on arrival to your unit? Yes/ No

Sepsis is SIRS (Systemic Inflammatory Response Syndrome) with a suspected or confirmed bacterial, viral, or fungal cause. SIRS is a response to a stimulus, which results in two or more of the following: temperature > 38.5°C or < 36°C, tachycardia (> two standard deviations above normal), or bradycardia in children < 1 year old (< 10th centile for age), tachypnea (> two standard deviations above normal), leukocyte count > 12,000 cells/mm3, < 4,000 cells/mm3, hyperglycemia, altered mental status, hyperlactemia, increased capillary refill time >2 seconds.

- Were broad spectrum antibiotics given at the time of diagnosis?

Yes/ No: not within 1 hour, but they were given on the first day of admission/ No: not given

Broad spectrum antibiotics cover gram negative, gram positive and anaerobic bacteria.

- Were broad spectrum antibiotics continued until at least 48 hours following surgery? Yes/ No
- If the neonate required ventilatory support at any stage during their perioperative care, was it available? Yes/ No/ Not applicable: not required
- Did the neonate have preoperative bowel perforation? Yes/ No
- On what day following admission did the neonate receive surgical intervention?

Please include the first day of admission as day 1. For example, for a neonate admitted on 2nd October with an anorectal malformation and receiving a stoma on 4th October, please insert 3 days. Enter 0 if no intervention was undertaken.

- What was the primary operation undertaken?

None, spontaneously stooling/ Dilatation of perineal or vestibular fistula/ Loop transverse colostomy/ Divided transverse colostomy/ Loop sigmoid colostomy/ Divided sigmoid colostomy/ Other stoma/ Anoplasty Posterior sagittal anorectoplasty (PSARP)/ Modified PSARP/ Abdomino-perineal anorectoplasty/ Other

If 'modified PSARP' or 'other', please provide details:

- If primary anorectal reconstruction was undertaken, was a Peña stimulator or equivalent device used to identify the position of the muscle complex intra-operatively?
- If primary anorectal reconstructive surgery was undertaken, did they have a covering stoma?

Yes/ No/ Not applicable

- Did the patient have any of the following complications within 30 days of surgery?

Electrolyte disturbance/ High output stoma (over 20mls/kg/day)/ Stoma prolapse/ Peri-stoma skin breakdown (or perianal if primary reconstructive surgery undertaken without a covering stoma)/ Sepsis/ Anal stenosis in those undergoing primary anorectal reconstruction without covering stoma

- What is the plan for future management?

No further operative management/ Anoplasty or pull-through planned at your hospital/ Anoplasty or pull-through planned at another hospital/ Stoma closure planned at your hospital/ Stoma closure planned at another hospital

Please tick all that apply.

Intussusception

- Age at presentation (in months):

Please round to the nearest month If born prematurely, please enter the number of months since birth.

- How was the intussusception diagnosed?

Clinically/ On ultrasound/ Other (please provide details). Tick all that apply

- Septic on arrival? Yes/ No

Sepsis is SIRS (Systemic Inflammatory Response Syndrome) with a suspected or confirmed bacterial, viral, or fungal cause. SIRS is a response to a stimulus, which results in two or more of the following: temperature > 38.5°C or < 36°C, tachycardia (> two standard deviations above normal), or bradycardia in children < 1 year old (< 10th centile for age), tachypnea (> two standard deviations above normal), leukocyte count > 12,000 cells/mm3, < 4,000 cells/mm3, hyperglycemia, altered mental status, hyperlactemia, increased capillary refill time >2 seconds.

- Appropriate antibiotics given within an hour of diagnosis?

Yes/ No, not within one hour, but they were given prior to intervention (or death)/ No, not given

Appropriate antibiotics include those that cover common anaerobic and aerobic gut organisms.

- If hypovolaemic or septic, were intravenous fluids administered prior to the primary intervention?

Hypovolaemia criteria include: prolonged capillary refill time >2 seconds, and/ or tachycardia (> two standard deviations above normal for age), mottled skin, reduced urine output (< 1ml/kg/hr), cyanosis, impaired consciousness, hypotension (mean arterial pressure < 5th

percentile for age). Please select 'yes' only if 10ml/kg or more of intravenous fluid was administered.

- Did the patient have a contraindication to air- or hydro-enema reduction?

Yes, peritonitis/ Yes, perforation/ Yes, non-responsive shock/ Yes, other/ No

- What primary management did the patient have?

None attempted (palliative care)/ Air-enema reduction without anaesthetic/ Air-enema reduction with anesthetic/ Hydro-enema reduction without anesthetic/ Hydro-enema reduction with anesthetic/ Laparotomy/ Laparoscopy/ Other (please specify)

If laparoscopy was used, was it converted to open?

- If air or enema reduction was attempted primarily, was it successful?

Yes/ No/ Not applicable, not attempted.

- How many attempts did it take to reduce the intussusception?

One attempt is defined as a series of increasing pressures, commonly up to a maximum of 120mmHg. A second attempt is considered to be a separate episode starting at the lowest pressure again.

- If air- or hydro-enema reduction was attempted primarily, was there a complication?

No/ Yes, perforation/ Yes, cardiorespiratory compromise and deterioration/ Yes, other

- If surgical intervention was required, what procedure was undertaken?

Manual reduction only/ Bowel resection and primary anastomosis/ Bowel resection and stoma

- How many hours following admission was the intussusception successfully reduced?

Please round to the nearest hour. If air- or hydro-enema reduction were attempted, but were unsuccessful, then the child had the intussusception reduced at laparotomy, please include the number of hours from admission to reduction in the operating room.

- Recurrence of intussusception within 30 days of original reduction?

Yes, within 30-days of original reduction/ No (patient not followed-up past discharge)/ No (patient followed-up at 30-days post original reduction)

Appendicitis

- Age at diagnosis (in years):

Please round to the nearest whole year.

- Septic on arrival? Yes/ No

Sepsis is SIRS (Systemic Inflammatory Response Syndrome) with a suspected or confirmed bacterial, viral, or fungal cause. SIRS is a response to a stimulus, which results in two or more of the following: temperature > 38.5°C or < 36°C, tachycardia (> two standard deviations above normal), or bradycardia in children < 1 year old (< 10th centile for age), tachypnea (> two standard deviations above normal), leukocyte count > 12,000 cells/mm3, < 4,000 cells/mm3, hyperglycemia, altered mental status, hyperlactemia, increased capillary refill time >2 seconds.

- Appropriate pre-operative antibiotics given at the time of diagnosis?

Yes/ No, not within 1 hour, but they were given pre-operatively or on the day of admission if managed conservatively/ No, not given pre-operatively or on the day of admission

Appropriate antibiotics include those that cover common anaerobic and aerobic gut organisms.

- If hypovolaemic or septic, was intravenous fluid resuscitation administered pre-operatively?

Yes/ No/ Not applicable - not hypovolemic or septic

Hypovolaemia criteria include: prolonged capillary refill time >2 seconds, and/ or tachycardia (> two standard deviations above normal for age), mottled skin, reduced urine output (< 1ml/kg/hr), cyanosis, impaired consciousness, hypotension (mean arterial pressure < 5th percentile for age). Please select 'yes' only if 10ml/kg or more of intravenous fluid was administered.

- How many hours following admission was the surgery undertaken?

Please round to the nearest hour.

- What time of day was the operation started (to the nearest hour)?
- Operation undertaken:

Open appendicectomy (Lanz or Gridiron)/ Open appendicectomy (Laparotomy, midline incision or other)/ Laparoscopic appendicectomy/ Open right hemicolectomy/ Laparoscopic right hemicolectomy/ Appendix mass palpated pre-operatively, surgery not undertaken/ Appendix mass palpated under anesthesia, surgery not undertaken/ No appendix mass palpated, patient managed conservatively with antibiotics

Other (please specify)

Conversion to open procedure? Yes/ No

- Operation findings:

Simple appendicitis/ Perforated appendicitis

If the appendix was gangrenous, please tick perforated.

- Was the peritoneal cavity washed out prior to closure?
- If yes, what was used to wash it out?
- Duration of appropriate intra-venous post-operative antibiotics (in days):

If no intravenous antibiotics were given, please enter 0. If the patient was managed conservatively, please enter the total number of days of intravenous antibiotics given.

- Duration of appropriate oral antibiotics given post-operatively (in days):

Please round to the nearest day. If intravenous antibiotics were given primarily then changed to oral antibiotics, please just enter the number of days of oral antibiotics here. If the patient was managed conservatively, enter the total number of days of oral antibiotics given.

- Did the patient have a post-operative intra-abdominal collection?

No/ Yes, drained percutaneously with no imaging/ Yes, drained percutaneously with ultrasound guidance/ Yes, drained percutaneously with CT guidance/ Yes, drained via laparoscopy/ Yes, drained via a second open operation/ Yes, requiring a prolonged antibiotic course but no further surgical intervention/ Not applicable, not surgical intervention undertaken

- If yes, how was the intra-abdominal collection diagnosed?

Clinically/ On ultrasound/ On CT/ At operation/ Other

- If visualised on imaging or at operation, was the collection 5cm in diameter or greater?

Inguinal hernia

- Age at the time of operation (in weeks):

Please round to the nearest week. For preterm neonates please input the number of weeks they have been alive since birth.

- Hernia type (at the time of operation):

Easily reducible/ Reduced with difficulty/ Irreducible or obstructed or strangulated/ Fistulated

- Unilateral or bilateral?
- Operation type: Elective/ Emergency
- If emergency, how many hours from admission to primary surgery?

Please round to the nearest hour.

- Operation technique:

Open herniotomy/ Laparoscopic herniotomy/ Laparotomy/ Other
If other, please provide details:

- Was there a conversion from laparoscopic to open?
- Was a bowel resection required at the time of surgery?

No/ Yes, via the inguinal incision with primary anastomosis/ Yes, via laparotomy with primary anastomosis/ Yes, with stoma formation/ Yes, other operation performed (please specify)

- Was the testis found to be gangrenous at the time of surgery? Yes/ No
- Were broad spectrum prophylactic antibiotics given at the time of the operation? Yes/ No

Broad spectrum antibiotics cover gram negative, gram positive and anaerobic bacteria.

- Duration of broad spectrum antibiotics given following surgery (in days):

If no antibiotics were given following surgery, please enter 0.

- Was there a recurrence of the hernia within 30 days of primary surgery?

Yes/ No: not followed up after discharge/ No: not at 30-day follow-up in person/ No: not at 30-day follow-up via telephone/ No: not at 30-day follow-up via another means

- Was the patient kept in overnight for continuous apnoea and saturation monitoring?

Yes/ No: discharged the same day/ No: kept overnight but not monitored

Appendix Methods 2. Hospital Survey

Title:
Surname/ Last Name:
First name:
Professional position:
Specialty:
Full name of institution:
Address of institution:
Country:

Personnel:

Number of Consultant Paediatric Surgeons undertaking general pediatric surgery at your institution:
Number of Consultant Paediatric Surgeons undertaking neonatal surgery at your institution:
Number of Consultant General Surgeons (covering adults and children) undertaking general paediatric surgery at your institution:
Number of Consultant General Surgeons (covering adults and children) undertaking neonatal surgery at your institution:
Number of medical officers undertaking general paediatric surgery independently at your institution:
Number of medical officers undertaking neonatal surgery independently at your institution:
Population served by your institution (in millions, including children and adults):

Infrastructure:
Please state whether the following facilities are available at your institution when required (Each field requires an answer: always, sometimes or never available):

Running water
Electricity
Electricity generator back-up
Laboratory for biochemistry
Laboratory for hematology
Blood bank
Functioning ultrasound (US) machine
Fluoroscopy
Paediatric ventilation outside the operating room (OR)
Neonatal ventilation outside the OR
Paediatric intensive care unit for surgical pediatric patients pre and post operatively if required
Neonatal intensive care unit for surgical neonates pre and post operatively (including if a stoma is present)
Parenteral nutrition
Surgical Safety Checklist in the OR
Sterile gloves and gown
Autoclave for sterilising surgical equipment
Peña stimulator or equivalent device to identify the muscle complex during anorectal reconstruction

---

Procedures:
Please state whether the following procedures are available at your institution when clinically appropriate/ required (Each field requires an answer: always, sometimes or never available):

Bedside primary reduction and closure of gastroschisis (Bianchi technique)
Preformed silo application, reduction and closure of gastroschisis
Surgical silo application, reduction and closure of gastroschisis
Primary closure of gastroschisis in the operating room
Sigmoid colostomy for anorectal malformation
Posterior Sagittal Anorectoplasty (PSARP) for anorectal malformation
Open appendicectomy
Laparoscopic appendicectomy
Ultrasound guided drainage of intra-abdominal collection
CT guided drainage of intra-abdominal collection
Open inguinal herniotomy

. *BMJ Global Health* 2021; 6:e004406. doi: 10.1136/bmjgh-2020-004406

Laparoscopic inguinal herniotomy
Radiologist trained to diagnose intussusception on ultrasound
Air-enema reduction of intussusception
Hydro-enema reduction of intussusception
Laparotomy for intussusception
Paediatric central line insertion
Neonatal central line insertion
Umbilical vein catheterisation

Anesthesia and resuscitation:
Please state whether the following facilities are available at your institution when required (Each field requires an answer: always, sometimes or never available):

Bottled oxygen
Piped oxygen
Oxygen saturation monitor
Apnea monitor
Multi-parameter intra-operative monitoring
Paediatric bag, valve and mask
Anaesthetic machine for neonates
Anaesthetic machine for children
Ketamine anaesthesia for children
Ketamine anaesthesia for neonates
Spinal/ caudal anaesthesia for children
Spinal/ caudal anaesthesia for neonates
Anaesthetic doctor competent to perform paediatric anesthesia
Anaesthetic doctor competent to perform neonatal anesthesia
Anaesthetic nurse competent to perform paediatric anesthesia
Anaesthetic nurse competent to perform neonatal anesthesia

Does your country have at least one children's hospital that can provide neonatal and paediatric surgery?

Any other comments:

Appendix Methods 3. Power calculation

Estimated patient numbers per hospital during a one month study period were: 1-2 with gastroschisis[1-3], 1-2 with anorectal malformation[4-7], 1 with intussusception[8], 11 with appendicitis[9,10] and 14 with an inguinal hernia[11-17] (29 patients per centre). Estimates were calculated using the mean number of patients presenting per month to hospitals in sub-Saharan Africa (SSA) who have published data on these conditions as referenced below.

Power calculations were undertaken to determine the mortality rate required in SSA for there to be a significant difference between SSA and published high-income country (HIC) benchmark data if 50 SSA hospitals participated in the study (1450 patients). The minimum differences that can be detected at a 5% significance level with 80% power are summarized in the table below.

The minimum detectable differences in mortality between SSA and HICs if 50 hospitals participated in the study

	High income country		Sub-Saharan Africa	
	Patient population*	Mortality rate*	Estimated study population size	Mortality rate in SSA at minimum detectable difference
**Gastroschisis**	301	4%	75	14.5%
**ARM**	410	3%	75	12.2%
**Intussusception**	9186	0.2%	50	5%
**Appendicitis**	24665	0.004%	550	0.43%
**Inguinal hernia**	10137	0%	700	0.40%

* sources of benchmark data are detailed in Appendix Table 1.

All mortality rates at the minimum detectable difference are lower than previously reported in SSA suggesting at 50 hospitals there should be adequate power to detect significant differences between observed SSA data and benchmark HIC data.

References

1. Agugua NE, Nwako FA. Gastroschisis a fifteen-year experience. West Afr J Med, 1990;9(2):147-50.
2. Arnold M. Is the incidence of gastroschisis rising in South Africa in accordance with international trends? A retrospective analysis at Pretoria Academic and Kalafong Hospitals, 1981-2001. S Afr J Surg, 2004;42(3):86-8.
3. Wright NJ, Zani A, Ade-Ajayi N. Epidemiology, management and outcome of gastroschisis in Sub-Saharan Africa: Results of an international survey. Afr J Paediatr Surg, 2015;12(1):1-6.
4. Govender S, Wiersma R. Delayed diagnosis of anorectal malformations (ARM): causes and consequences in a resource-constrained environment. Pediatr Surg Int, 2016;32(4):369-75.
5. Kigo CN, Ndung'u JM. Bowel function following primary repair of anorectal malformations at Kenyatta National Hospital. East Afr Med J, 2002;79(3):124-7.
6. Chirdan LB, Uba FA, Ameh EA, Mshelbwala PM. Colostomy for high anorectal malformation: an evaluation of morbidity and mortality in a developing country. Pediatr Surg Int, 2008;24(4):407-10.
7. Lawal TA, Olulana DI, Ogundoyin OO. Spectrum of colorectal surgery operations performed in a single paediatric surgery unit in sub-Saharan Africa. Afr J Paediatr Surg, 2014;11(2):128-31.
8. Jiang J, Jiang B, Parashar U, et al. Childhood intussusception: a literature review. PLoS One, 2013;8(7):e68482.
9. Derbew M, Ahmed E. The pattern of pediatric surgical conditions in Tikur Anbessa Unversity Hospital, Addis Ababa, Ethiopia. Ethiop Med J, 2006;44(4):331-8.
10. Kong VY, Sartorius B, Clarke DL. Acute appendicitis in the developing world is a morbid disease. Ann R Coll Surg Engl, 2015;97(5):390-5.
11. Bamigbola KT, Nasir AA, Abdur-Rahman LO, Adeniran JO. Complicated childhood inguinal hernias in UITH, Ilorin. Afr J Paediatr Surg, 2012;9(3):227-30.
12. Eeson G, Birabwa-Male D, Pennington M, Blair GK. Costs and cost-effectiveness of pediatric inguinal hernia repair in Uganda. World J Surg, 2015;39(2):343-9.
13. Zamakhshary M, To T, Guan J, Langer JC. Risk of incarceration of inguinal hernia among infants and young children awaiting elective surgery. CMAJ, 2008;179(10):1001-5.
14. Ameh EA. Morbidity and mortality of inguinal hernia in the newborn. Niger Postgrad Med J, 2002;9(4):233-4.
15. Usang UE, Sowande OA, Adejuyigbe O, Bakare TI, Ademuyiwa OA. The role of preoperative antibiotics in the prevention of wound infection after day case surgery for inguinal hernia in children in Ile Ife, Nigeria. Pediatr Surg Int, 2008;24(10):1181-5.
16. Ameh EA. Incarcerated and strangulated inguinal hernias in children in Zaria, Nigeria. East Afr Med J, 1999;76(9):499-501.
17. Ohene-Yeboah M, Abantanga F, Oppong J, et al. Some aspects of the epidemiology of external hernias in Kumasi, Ghana. Hernia, 2009;13(5):529-32.

Appendix Figure 1. Additional hospitals providing survey data without patient data (n=23)

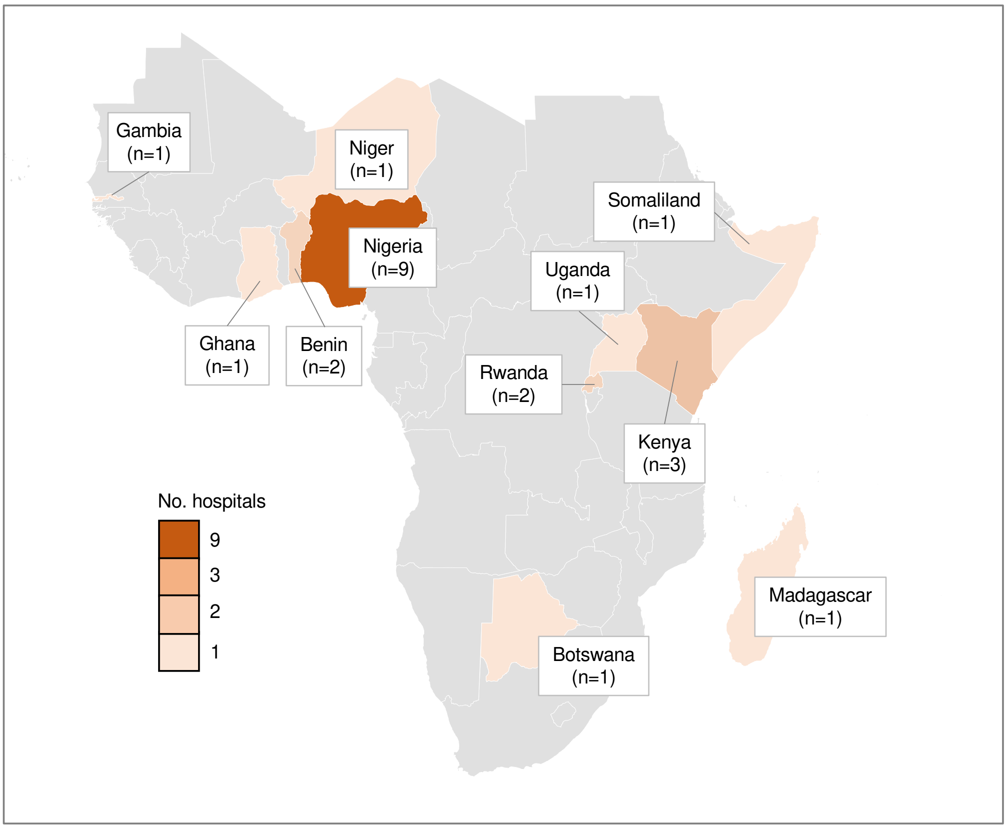

. *BMJ Global Health* 2021; 6:e004406. doi: 10.1136/bmjgh-2020-004406

Appendix Table 1. High-income country benchmark data used as a comparator to observed SSA data

Condition	Study(s) used as comparator*	Study region, type and population.	Mortality rate, % (deaths/study population)	Timeframe of mortality rate and comparability to PaedSurg Africa study results
Gastroschisis	Bradnock et al, 2011[1]	UK. National cohort study, whole population.	2.0% (6/302)	Neonatal period and within 30-days of primary intervention (equivalent to the PaedSurg Africa study). Of note, the study reports an overall mortality of 4.0% (12/302) at 1-year, but 6/302 deaths were after 30-days from primary intervention and hence were not included within the benchmark HIC mortality used as a comparator.
ARM	Rintala et al, 2008[2]	Finland. Single author's case series (270 high ARM, 140 low ARM)	2.9% (12/410)	The author describes the mortality timeframe within 'short-term outcomes' prior to 'early childhood'. Hence, the timeframe could be longer than in the PaedSurg Africa study. Also, the data is from 1984-1998 and hence outcomes could have improved further since. Overall, this mortality rate could be an overestimation of the current mortality from ARM in HICs, but it is unlikely to be an underestimation. Also, the Rintala series has a higher proportion of patients with high ARM (65.9%) than this study (50.5%) and high ARM is associated with higher mortality. Hence, again this difference in the cohorts could result in an underestimation of the disparity in mortality between SSA and HICs, but unlikely an overestimation.
Intussusception	Jiang et al, 2013[3]	Australia, Europe, New Zealand, USA. Literature review of studies published globally since 2002. Data from 34 studies: 11 national, 9 regional, 14 hospital.	0.2% (18/9186)	Mortality was defined as 'Case-fatality among children hospitalised with intussusception'. Hence, this is equivalent to the all-cause, in-hospital mortality utilised in this study and indeed the 30-day post-intervention mortality since no deaths were reported following discharge.
Appendicitis	Healy et al, 2015[4]	Canada, UK, USA. Systematic review and meta-analysis. Data for mortality were pooled from 3 hospital based studies containing both paediatric and general surgeon operators.	0.004% (1/24665)	One death occurred during the primary hospital admission. This is comparable with the data from this study which also had one death during the primary admission.
Inguinal hernia†	Ein et al, 2006[5]	Canada. Single operator case series (n=6361) from 1969 to 2004.	0% (0/6361)	1-12 month follow-up (mean 6-months). Hence, the mortality at discharge and 30-day post primary intervention follow-up was 0% to use as a comparator.

* These represent the published HIC studies with the largest sample sizes and most representative populations at the time of study design. †The Erdogan et al study detailed as a comparator in the PaedSurg Africa study protocol was not used as it was based in Turkey, which is upper middle income rather than high-income. ARM: Anorectal malformation. HIC: High-income country. SSA: Sub-Saharan Africa. UK: United Kingdom. USA: United States of America.

References

1. Bradnock TJ, Marven S, Owen A, et al; BAPS-CASS. Gastroschisis: one year outcomes from national cohort study. BMJ. 2011;343:d6749.
2. Rintala R, Pakarinen M. Imperforate anus: long- and short-term outcome. Sem Pediatr Surg, 2008;17:79-89.
3. Jiang J, Jiang B, Parashar U, et al. Childhood intussusception: a literature review. PLoS One, 2013;8(7):e68482.
4. Healy DA, Doyle D, Moynagh E, et al. Systematic review and meta-analysis on the influence of surgeon specialization n outcomes following appendicectomy in children. Medicine (Baltimore), 2015;94(32):e1352.
5. Ein SH, Njere I, Ein A. Six thousand three hundred sixty-one pediatric inguinal hernias: a 35-year review. J Pediatr Surg, 2006;41(5):980-6.

Appendix Table 2a. Condition specific patient characteristics, management and outcomes for patients with gastroschisis, n=111 (Data from 36 hospitals)

Variable	Number of cases, n (%)
**Type of gastroschisis:**	
Simple	80 (72.1)
Complex with atresia	5 (4.5)
Complex with necrosis	22 (19.8)
Complex with perforation	3 (2.7)
Complex with atresia and perforation	1 (0.9)
**Was it diagnosed antenatally?**	
No	106 (95.5)
Yes	5 (4.5)
**Septic on arrival:**	
No	50 (45.5)
Yes	60 (54.5)
Missing*	1
**Broad spectrum antibiotics given within an hour of presentation?**	
Yes	56 (50.5)
Not within 1hr, but on the first day of admission	53 (47.7)
Not on the first day of admission	2 (1.8)
**Were broad spectrum antibiotics continued for 48-hours after abdominal wall closure?**	
No	22 (23.9)
Yes	70 (76.1)
NA - palliative care*	19
**If the neonate was hypothermic, were they warmed?**	
No	19 (22.6)
Yes	65 (77.4)
NA - not hypothermic*	27
**Was the neonate hypovolaemic at presentation?**	
No	47 (42.3)
Yes	64 (57.7)
**Did the neonate receive an intravenous fluid bolus within an hour of presentation?**	
Yes	58 (52.7)
Not within 1hr, but on the first day of admission	41 (37.3)
Not on the first day of admission	11 (10.0)
Missing*	1
**Peripheral cannula on the first day:**	
No	7 (6.3)
Yes	104 (93.7)
**Umbilical vein catheter (UVC) on the first day:**	
No	104 (93.7)
Yes	7 (6.3)
**Central line (excluding UVC) on the first day:**	
No	106 (95.5)
Yes	5 (4.5)
**Time from admission to primary intervention (hours). Median, IQR (range)†**	**7**, 3-18, (0-168)
**Method of gastroschisis closure:**	
Not attempted - palliative care	20 (18.2)
Primary closure at the cotside (Bianchi technique)	5 (4.5)
Preformed silo, reduction and closure at cotside	33 (30.0)
Preformed silo, reduction and closure in the operating room	13 (11.8)
Surgical silo, reduction and closure in the operating room	8 (7.3)
Primary closure in the operating room	16 (14.5)
Improvised silo with plastic or female condom	15 (13.6)
Missing*	1
**On what day following admission was abdominal wall closure achieved? Median, IQR (range)†**	1, 0-3, (0-48)

Variable	Number of cases, n (%)
**If the neonate required ventilatory support, was it available?**	
No	36 (50.7)
Yes	35 (49.3)
NA - not required*	40
**Did the neonate receive parenteral nutrition?**	
No	70 (63.1)
Yes, but for less time than required	15 (13.5)
Yes, until full enteral intake was established	26 (23.4)
**Did the neonate develop bowel ischaemia following intervention?**	
No	60 (70.6)
Yes	25 (29.4)
NA*	24
Missing*	2
**Did the neonate develop following intervention?**	
No	54 (85.7)
Yes	9 (14.3)
NA*	43
Missing*	5
**If abdominal compartment syndrome developed, was the abdomen re-opened?**	
No	6 (66.7)
Yes	3 (33.3)
**Did the neonate develop central line sepsis?**	
No	40 (90.9)
Yes	4 (9.1)
NA*	62
Missing*	5

* Excluded from percentage calculations. † One patient was missing data for each of these variables. IQR: Interquartile range. NA: Not applicable. Percentages have been rounded and may not total 100.

Appendix Table 2b. Condition specific patient characteristics, management and outcomes for patients with anorectal malformation, n=188 (Data from 42 hospitals)

Variable	Number of cases, n (%)
**Type of anorectal malformation:**	
Low	92 (49.5)
High	94 (50.5)
Missing*	2
**Additional congenital anomaly:**	
No	136 (72.3)
Yes	52 (27.7)
**Vertebral anomaly:**	
No	180 (95.7)
Yes	8 (4.3)
**Cardiac anomaly:**	
No	176 (93.6)
Yes	12 (6.4)
**Oesophageal atresia:**	
No	186 (98.9)
Yes	2 (1.1)
**Tracheo-oesophageal fistula:**	
No	185 (98.4)
Yes	3 (1.6)
**Renal anomaly:**	
No	184 (97.9)
Yes	4 (2.1)
**Limb anomaly:**	
No	180 (95.7)
Yes	8 (4.3)
**Other anomaly:**	
No	158 (84.0)
Yes	30 (16.0)
**Septic on arrival?**	
No	146 (78.1)
Yes	41 (21.9)
Missing*	1
**Broad spectrum antibiotics within an hour of presentation?**	
Yes	60 (32.4)
Not within an hour, but on the first day	65 (35.1)
Not on the first day of presentation	60 (32.4)
Missing*	3
**Broad spectrum antibiotics for 48-hours postoperatively?**	
No	31 (17.8)
Yes	143 (82.2)
Missing*	14
**If ventilatory support was required, was it available?**	
No	60 (63.8)
Yes	34 (36.2)
NA, not required*	88
Missing*	6
**Preoperative bowel perforation:**	
No	179 (96.2)
Yes	7 (3.8)
Missing*	2
**Time from admission to primary intervention (days). Median, IQR (range)†**	2, 1.5-4, (0-607)
**Operation undertaken:**	
None, palliative care	7 (3.8)

. *BMJ Global Health* 2021; 6:e004406. doi: 10.1136/bmjgh-2020-004406

None, spontaneous stooling	8 (4.3)
Anal dilatation only	11 (5.9)
Loop transverse colostomy	9 (4.9)
Divided transverse colostomy	6 (3.2)
Loop sigmoid colostomy	20 (10.8)
Divided sigmoid colostomy	77 (41.6)
Other stoma	1 (0.5)
Anoplasty	17 (9.2)
Posterior Sagittal Anorectoplasty (PSARP)	27 (14.6)
Abdominoperineal Anorectoplasty (APARP)	1 (0.5)
Modified Posterior Sagittal Anorectoplasty (PSARP)	1 (0.5)
Missing*	3
**If primary anorectal reconstruction was undertaken, was the patient given a covering stoma?**	32 (71.1)
No	13 (28.9)
Yes	1
Missing*	
**If anorectal reconstruction was undertaken, was a Peña stimulator or equivalent used to locate the sphincter complex?**	
No	5 (10.9)
Yes	23 (50.0)
No, available but not used	2 (4.3)
No, not available	16 (34.8)
**Electrolyte disturbance within 30 days of surgery?**	
No	128 (83.1)
Yes	26 (16.9)
NA*	26
Missing*	8
**High stoma output above 20mls/kg/day within 30 days of surgery?**	
No	120 (97.6)
Yes	3 (2.4)
NA*	62
Missing*	3
**Stoma prolapse within 30 days of surgery?**	
No	118 (95.9)
Yes	5 (4.1)
NA*	62
Missing*	3
**Peri-stoma skin breakdown within 30 days of surgery?**	
No	100 (82.6)
Yes	21 (17.4)
NA*	62
Missing*	5
**Sepsis within 30 days of surgery?**	
No	132 (85.7)
Yes	22 (14.3)
NA*	26
Missing*	8
**Anal stenosis within 30 days of surgery in those with primary anorectal reconstruction?**	
No	39 (84.8)
Yes	7 (15.2)
NA*	142
**Plan for future surgical intervention:‡**	
Anoplasty/ pull-through at current centre	118 (62.8)
Anoplasty/ pull-through elsewhere	7 (3.7)
Stoma closure at current hospital	47 (25.0)
Stoma closure elsewhere	0 (0.0)
No further surgery planned	34 (18.1)

* Excluded from percentage calculations. † Missing data, n=5. ‡ Some patients had more than one future planned intervention; total number of patients used as denominator. NA: Not applicable. Percentages have been rounded and may not total 100.

Appendix Table 2c. Condition specific patient characteristics, management and outcomes for patients with intussusception, n=225 (data from 42 hospitals)

Variable	Number of cases, n (%)
**Diagnosis of intussusception:**	
Clinically	40 (17.8)
Ultrasound	175 (77.8)
Other	10 (4.4)
**Septic on arrival?**	
No	123 (54.7)
Yes	102 (45.3)
**Appropriate antibiotics given within an hour of diagnosis?**	
Yes	132 (58.7)
Not within an hour, but prior to intervention	82 (36.4)
No, not prior to intervention	11 (4.9)
**If hypovolemic or septic, were intravenous fluids administered prior to intervention?**	
No	20 (11.4)
Yes	156 (88.6)
NA - not hypovolemic or septic*	48
Missing*	1
**Peritonitis as a contraindication for air- or hydro-enema reduction:**	
No	184 (81.8)
Yes	41 (18.2)
**Perforation as a contraindication for air- or hydro-enema reduction:**	
No	209 (92.9)
Yes	16 (7.1)
**Shock as a contraindication for air- or hydro-enema reduction:**	
No	213 (94.7)
Yes	12 (5.3)
**Other contraindication for air- or hydro-enema reduction:**	
No	167 (74.2)
Yes	58 (25.8)
**Contraindication for air- or hydro-enema reduction:**	
No	107 (47.6)
Yes	118 (52.4)
**Management:**	
Palliative care	7 (3.1)
Air enema reduction	5 (2.2)
Hydroenema reduction	53 (23.6)
Primary laparotomy	158 (70.2)
Spontaneous resolution	2 (0.9)
**If air- or hydro-enema reduction were used primarily, was it successful?**	
No	18 (31.0)
Yes	40 (69.0)
**If air- or hydro-enema reduction were used primarily, was there a complication?**	
No	53 (91.4)
Yes, perforation	3 (5.2)
Yes, other	2 (3.5)
**If surgical intervention was required, what intervention was undertaken?**	
Manual reduction	63 (35.6)
Bowel resection and primary anastomosis	107 (60.5)
Bowel resection and stoma	7 (4.0)
NA*	48
**Recurrence within 30-days:**	
Yes	4 (1.8)
No (followed-up to 30-days)	46 (20.7)
No, but not followed-up post-discharge	172 (77.5)
Missing*	3

* Excluded from percentage calculations. NA: Not applicable. Percentages have been rounded and may not total 100.

Appendix Table 2d. Condition specific patient characteristics, management and outcomes for patients with appendicitis, n=250 (Data from 41 hospitals)

Variable	Number of cases, n (%)
**Septic on arrival?**	
No	110 (44.0)
Yes	140 (56.0)
**Appropriate antibiotics given within an hour of diagnosis?**	
Yes	152 (60.8)
Not within an hour, but on the first day	96 (38.4)
Not on the first day of admission	2 (0.8)
**If hypovolemic or septic, were intravenous fluids administered preoperatively?**	
No	13 (7.7)
Yes	155 (92.3)
NA*	82
**Time from admission to surgery (hours). Median, IQR (range)**	12, 6-24 (0-150)
**Time of operation (24 hour clock):**	
Between 8am to midnight	205 (84.7)
Between midnight to 8am	37 (15.3)
Missing*	8
**Management:**	
Open appendicectomy (Lanz/ Gridiron)	163 (65.2)
Laparotomy	55 (22.0)
Laparoscopic appendicectomy	25 (10.0)
Open right hemicolectomy	1 (0.4)
Non-operative, mass palpated preoperatively	1 (0.4)
Non-operative, mass palpated under anesthetic	2 (0.8)
Other	3 (1.2)
**Conversion to open from laparoscopic:**	
No	24 (96.0)
Yes	1 (4.0)
**Operation findings:**	
Simple appendicitis	105 (42.9)
Perforated appendicitis	140 (57.1)
NA*	3
Missing*	2
**Peritoneal cavity washout prior to closure?**	
No	89 (36.3)
Yes	156 (63.7)
NA*	5
**Washout used:**	
Normal saline	141 (95.9)
Betadine	2 (1.4)
Other	4 (2.7)
NA - no washout	89
Missing*	14
**Duration of postoperative intravenous antibiotics (days). Median, IQR (range)**	3, 2-5 (0-35)
**Duration of postoperative oral antibiotics (days). Median, IQR (range)**	5, 2-7 (0-36)
**Postoperative intra-abdominal collection:**	
No	225 (90.4)
Yes, requiring antibiotics only	5 (2.0)
Yes, percutaneous drainage without imaging	2 (0.8)
Yes, surgical drainage	14 (5.6)
NA	3 (1.2)
Missing*	1

Variable	Number of cases, n (%)
**If yes, how was the collection diagnosed?**	
Clinically	7 (33.3)
Ultrasound	13 (61.9)
CT	1 (4.8)
**If the collection was visualised on imaging or at operation, was it above 5cm in diameter?**	4 (28.6)
No	10 (71.4)
Yes	

* Excluded from percentage calculations. CT: Computer tomography. IQR: Interquartile range. NA: Not applicable.

. *BMJ Global Health* 2021; 6:e004406. doi: 10.1136/bmjgh-2020-004406

Appendix Table 2e. Condition specific patient characteristics, management and outcomes for patients with inguinal hernia, n=633 (Data from 50 centres)

Variable	Number of cases, n (%)
**Type of hernia:**	
Easily reducible	532 (84.2)
Reduced with difficulty	41 (6.5)
Incarcerated, obstructed or strangulated	57 (9.0)
Fistulated	2 (0.3)
Missing*	1
**Unilateral**	561 (88.6)
**Bilateral**	72 (11.4)
**Operation type:**	
Elective	570 (90.5)
Emergency	63 (9.5)
**Operation undertaken:**	
Open herniotomy	619 (97.8)
Laparoscopic herniotomy	5 (0.8)
Laparotomy	7 (1.1)
Other	2 (0.3)
**Conversion from laparoscopic to open?**	
No	5 (100.0)
Yes	0 (0.0)
**Bowel resection required?**	
No	616 (97.3)
Yes, with primary anastomosis via inguinal incision	7 (1.1)
Yes, with primary anastomosis via laparotomy	8 (1.3)
Yes, requiring a stoma	1 (0.2)
Yes, other	1 (0.2)
**Testis gangrenous at the time of operation?**	
No	615 (97.5)
Yes	16 (2.5)
Missing*	2
**Broad spectrum antibiotics given at the time of operation:**	
No	379 (60.0)
Yes	253 (40.0)
Missing*	1
**Duration of broad spectrum antibiotics given following surgery (days). Median, IQR (range)†**	0, 0-2 (0-21)
**Recurrence within 30 days of surgery:**	
Yes	8 (1.3)
No, but no follow-up post-discharge	82 (13.1)
No, followed-up in person	405 (64.5)
No, followed-up via the telephone	93 (14.8)
No, other follow-up undertaken	40 (6.4)
Missing*	5
**Overnight stay at the hospital with apnoea and saturation monitoring?**	
No, same day discharge	160 (25.3)
Admitted, but not monitored	132 (20.9)
Yes	340 (53.8)
Missing*	1

* Excluded from percentage calculations. † Missing data, n=3. IQR: Interquartile range. Percentages have been rounded and may not total 100.

Appendix Table 3. Mortality stratified by age

Age	Neonate (0-28 days) % (no. deaths/ no. neonates)	Infant (29 – 364 days) % (no. deaths/ no. infants)	Child (1 – 16 years) % (no. deaths/ no. children)
**Gastroschisis**	75.5% (83/110)	0.0% (0/0)	0.0% (0/0)
**Anorectal malformation**	21.5% (28/130)	0.0% (0/37)	20.0% (4/20)
**Intussusception**	100.0% (1/1)	9.5% (18/189)	5.8% (2/34)
**Appendicitis**	0.0% (0/0)	0.0% (0/0)	0.4% (1/248)
**Inguinal hernia**	0.0% (0/26)	1.1% (2/177)	0.0% (0/418)
**All patients***	41.9% (112/267)	5.0% (20/403)	1.0% (7/720)

* Missing data, n=17 (Gastroschisis n=1, ARM n=1, Intussusception n=1, Appendicitis n=2, Inguinal hernia n=12)

Appendix Table 4. Patient follow-up

Variable	All conditions (n=1407), n (%)	Gastroschisis (n=111), n (%)	Anorectal malformation (n=188), n (%)	Intussusception (n=225), n (%)	Appendicitis (n=250), n (%)	Inguinal hernia (n=633), n (%)
**Was the patient alive at 30 days post-intervention?**						
Yes	1037 (73.7)	16 (14.4)	118 (62.8)	166 (73.8)	206 (82.4)	531 (83.9)
No (died in hospital)	127 (9.0)	83 (74.8)	21 (11.2)	21 (9.3)	1 (0.4)	1 (0.2)
No (died after discharge)	12 (0.9)	0 (0.0)	11 (5.8)	0 (0.0)	0 (0.0)	1 (0.2)
Not followed-up to 30 days	211 (15.0)	11 (9.9)	35 (18.6)	33 (14.7)	39 (15.6)	93 (14.7)
Missing	20 (1.4)	1 (0.9)	3 (1.6)	5 (2.2)	4 (1.6)	7 (1.1)
**Was the patient followed-up to 30 days post-intervention to assess for complications?***						
Yes	1030 (73.2)	78 (70.3)	108 (57.4)	152 (67.6)	190 (76.0)	502 (79.3)
No	358 (25.4)	26 (23.4)	75 (39.9)	72 (32.0)	58 (23.2)	127 (20.1)
Missing	19 (1.3)	7 (6.3)	5 (2.7)	1 (0.4)	2 (0.8)	4 (0.6)

* Or followed-up until death if prior to 30 days.

Appendix Table 5. Univariable and multivariable analysis of patient-level and hospital-level factors associated with mortality for patients with gastroschisis, anorectal malformation and intussusception combined (generic patient variables only)

	Alive (n=396), n (%)	Dead (n=125), n (%)	Univariable analysis		Multivariable analysis of patient variables		Multivariable analysis of patient and hospital variables	
			OR (95% CI)	P value	OR (95% CI)	P value	OR (95% CI)	P value
**Patient level variables**								
Condition: Gastroschisis	27 (6.8)	83 (66.4)	reference		reference		reference	
Condition: ARM	166 (41.9)	21 (16.8)	0.04 (0.02,0.07)	**<0.001**	0.07 (0.02,0.18)	**<0.001**	0.09 (0.03, 0.25)	**<0.001**
Condition: Intussusception	203 (51.3)	21 (16.8)	0.03 (0.02,0.06)	**<0.001**	0.03 (0.01,0.10)	**<0.001**	0.04 (0.01,0.14)	**<0.001**
Gestational age at birth, median (IQR)	38 (37,40)	37 (36,38)	0.67 (0.59,0.77)	**<0.001**	**	**	**	**
Male	229 (57.8)	67 (53.6)	0.96 (0.62,1.50)	0.87				
Weight at presentation in kg, median (IQR)	6.5 (3.2,8.0)	2.5 (2.0,3.0)	0.67 (0.60,0.75)	**<0.001**	1.00 (0.87,1.17)	0.952	1.01 (0.87,1.16)	0.926
Time to presentation in days, median (IQR)	3.0 (2.0,7.0)	1.5 (1.0,3.0)	0.86 (0.78,0.95)	**0.002**	0.97 (0.91,1.03)	0.313	0.97 (0.91,1.03)	0.273
Distance from home in km, median (IQR)	30 (14,81)	40 (15,140)	1.00 (1.00,1.00)	0.853				
Mode of transport: Ambulance/ health vehicle	59 (15.0)	43 (34.7)	reference		reference		reference	
Mode of transport: Patient's own	329 (83.7)	75 (60.5)	0.33 (0.18,0.60)	**<0.001**	0.73 (0.30,1.78)	0.482	0.67 (0.26,1.69)	0.393
ASA score: 1-2	278 (70.2)	31 (25.0)	reference		reference		reference	
ASA score: 3-5	98 (24.7)	82 (66.1)	9.34 (5.13,17.03)	**<0.001**	3.91 (1.74,8.79)	**0.001**	3.31 (1.44,7.63)	**0.005**
ASA score: NA, No intervention	20 (5.1)	11 (8.9)	6.65 (2.54,17.42)	**<0.001**	0.58 (0.10,3.34)	0.539	0.58 (0.10,3.43)	0.546
WHO checklist used: No	186 (47.0)	74 (59.2)	reference		reference		reference	
WHO checklist used: Yes	184 (46.5)	27 (21.6)	0.37 (0.20,0.67)	**0.001**	0.69 (0.3,1.59)	0.382	0.74 (0.31,1.74)	0.486
WHO checklist used: NA, no intervention	26 (6.6)	24 (19.2)	3.09 (1.49,6.43)	**0.002**	0.85 (0.15,4.78)	0.858	0.84 (0.15,4.75)	0.846
Anaesthetic, General Anaesthetic: Yes	311 (78.5)	48 (38.4)	reference		reference		reference	
Anaesthetic, General Anaesthetic: No	55 (13.9)	43 (34.4)	4.83 (2.63,8.88)	**<0.001**	1.11 (0.41,2.98)	0.833	1.23 (0.45,3.41)	0.685
Anaesthetic, NA, no intervention	30 (7.6)	34 (27.2)	9.86 (4.99,19.52)	**<0.001**	10.48 (2.07,52.97)	**0.004**	10.33 (1.98,53.94)	**0.006**
Anaesthetist: Doctor	240 (60.6)	36 (28.8)	reference		**	**	**	**
Anaesthetist: Non-doctor	102 (25.8)	49 (39.2)	3.08 (1.67,5.68)	**<0.001**	**	**	**	**
Anaesthetist: NA, no anaesthetic	54 (13.6)	40 (32.0)	7.35 (3.88,13.93)	**<0.001**	**	**	**	**
Blood transfusion: Yes	107 (27.4)	42 (36.2)	1.81 (1.08,3.04)	**0.024**	3.72 (1.59,8.68)	**0.002**	3.95 (1.70,9.19)	**0.001**
Surgical site infection: Yes	83 (21.2)	19 (15.8)	0.73 (0.40,1.33)	0.303				
Wound dehiscence: Yes	29 (7.4)	2 (1.7)	0.18 (0.04,0.82)	**0.027**	**	**	**	**
Further intervention: Yes	47 (12.0)	13 (11.0)	0.80 (0.39,1.64)	0.538				
**Hospital level variables**								
Paediatric surgeons/million children, mean (SD)	2.18 (0.84)	2.28 (0.83)	1.10 (0.73,1.65)	0.651				
Personnel: low	56 (15.8)	3 (2.7)	reference				**	**
Personnel: medium	255 (71.8)	96 (85.0)	6.41 (1.50,27.32)	**0.012**			**	**
Personnel: high	44 (12.4)	14 (12.4)	4.98 (0.97,25.57)	0.054			**	**

	Alive (n=396), n (%)	Dead (n=125), n (%)	Univariable analysis		Multivariable analysis of patient variables		Multivariable analysis of patient and hospital variables	
			OR (95% CI)	P value	OR (95% CI)	P value	OR (95% CI)	P value
Infrastructure: low	55 (14.2)	11 (9.4)	reference					
Infrastructure: medium	240 (62.2)	70 (59.8)	1.39 (0.50,3.91)	0.528				
Infrastructure: high	91 (23.6)	36 (30.8)	1.23 (0.37,4.13)	0.732				
Procedure: low	80 (20.7)	57 (48.7)	reference				reference	
Procedure: medium	268 (69.4)	47 (40.2)	0.29 (0.15,0.54)	**<0.001**			0.44 (0.18,1.05)	0.064
Procedure: high	38 (9.8)	13 (11.1)	0.54 (0.22,1.30)	0.167			0.65 (0.18,2.32)	0.509
Anaesthetic and resuscitation: low	54 (14.0)	11 (9.4)	reference					
Anaesthetic and resuscitation: medium	222 (57.5)	73 (62.4)	1.51 (0.54,4.22)	0.432				
Anaesthetic and resuscitation: high	110 (28.5)	33 (28.2)	1.36 (0.46,4.05)	0.578				

** Excluded from the multivariable model due to collinearity or low counts. ARM: Anorectal malformation. ASA: American Association of Anesthesiologists. CI: Confidence interval. IQR: Interquartile range. NA: Not applicable. OR: Odds ratio. SD: Standard deviation. WHO: World Health Organisation. Percentages have been rounded and may not total 100.

. *BMJ Global Health* 2021; 6:e004406. doi: 10.1136/bmjgh-2020-004406

Appendix Table 6. Univariable analysis of patient-level and hospital-level factors associated with mortality for patients with gastroschisis (generic and condition specific variables)

	Alive (n=27), n (%)	Dead (n=83), n (%)	Univariable analysis	
			OR (95% CI)	P value
**Patient level variables: *generic***				
Gestational age at birth, median (IQR)	38 (36,40)	36 (35,38)	*	*
Male sex	8 (30)	39 (47)	2.40 (0.86,6.74)	0.095
Weight in kg, mean (SD)	2.50 (0.46)	2.26 (0.53)	0.40 (0.14,1.10)	0.076
Time to presentation in days, median (IQR)	1 (1,1)	1 (1,2)	1.16 (0.75,1.79)	0.502
Distance from home in km, median (IQR)	60 (22,195)	58 (16,153)	1.00 (0.99,1.00)	0.472
Place of birth: Outborn	25 (93)	76 (93)	1.01 (0.17,6.19)	0.987
ASA score: 1-2	17 (63)	18 (22)	reference	
ASA score: 3-5	9 (33)	54 (66)	7.98 (1.70,37.40)	**0.008**
ASA score: NA, no intervention	1 (4)	10 (12)	9.79 (0.98,98.07)	0.052
WHO Checklist used: No	8 (30)	51 (61)	*	*
WHO Checklist used: Yes	17 (63)	14 (17)	*	*
WHO Checklist used: NA	2 (7)	18 (22)	*	*
Anaesthetic: General anaesthetic	16 (59)	20 (24)	reference	
Anaesthetic: No general anaesthetic	9 (33)	39 (47)	4.48 (1.02,19.66)	**0.047**
Anaesthetic: NA, no intervention	2 (7)	24 (29)	13.28 (2.04,86.24)	**0.007**
Anaesthetist: Doctor	13 (48)	15 (18)	reference	
Anaesthetist: Non-doctor	11 (41)	38 (46)	3.46 (0.86,13.91)	0.08
Anaesthetist: NA, no anaesthetic	3 (11)	30 (36)	12.16 (2.18,67.77)	**0.004**
Blood transfusion: No	9 (33)	16 (19)	0.51 (0.17,1.52)	0.226
Surgical site infection: Yes	6 (25)	14 (20)	0.68 (0.20,2.33)	0.536
Wound dehiscence: Yes	2 (8)	1 (1)	0.06 (0.00,1.26)	0.070
Further unplanned intervention: Yes	5 (21)	10 (14)	0.48 (0.12,1.97)	0.311
**Patient level variables: *condition specific***				
Gastroschisis type: Complex	3 (11)	28 (34)	4.39 (1.12,17.20)	**0.034**
Antenatal diagnosis: Yes	2 (7)	3 (4)	0.42 (0.05,3.25)	0.407
Septic at presentation: Yes	12 (44)	48 (59)	2.33 (0.76,7.15)	0.140
Warmed if hypothermic on arrival: No	4 (15)	14 (17)	reference	
Warmed if hypothermic on arrival: Yes	15 (56)	50 (60)	0.99 (0.23,4.18)	0.989
Warmed if hypothermic on arrival: NA	8 (30)	19 (23)	0.58 (0.12,2.72)	0.487
Hypovolaemic on arrival: Yes	9 (33)	55 (66)	7.84 (1.96,31.34)	**0.004**
IV bolus within 1 hour of arrival: Yes	12 (46)	46 (55)	1.87 (0.64,5.50)	0.253
Peripheral cannula day 1: Yes	24 (89)	79 (95)	2.79 (0.49,15.80)	0.245
Umbilical catheter day 1: Yes	2 (7)	5 (6)	0.63 (0.09,4.25)	0.639
Other central line day 1: Yes	3 (11)	2 (2)	0.17 (0.02,1.46)	0.106
Time to intervention in hours, median (IQR)	6.5 (2.3,15.8)	7.5 (4.0,16.5)	1.00 (0.98,1.02)	0.820
Intervention: primary closure at the cotside or in the OR or surgical silo at the cotside or OR	22 (85)	35 (42)	*	*
Intervention: preformed silo at the cotside	4 (15)	29 (35)	*	*
Intervention: palliative care	0 (0)	19 (22)	*	*
Days to closure, median (IQR)	2 (1,5)	0 (0,2)	0.90 (0.77,1.04)	0.145
Ventilation available when required: No	10 (37)	25 (30)	reference	
Ventilation available when required: Yes	8 (30)	27 (33)	0.89 (0.20,3.96)	0.876
Ventilation available: NA, not required	9 (33)	31 (37)	0.99 (0.26,3.88)	0.994
Parenteral nutrition received: Yes	11 (41)	30 (36)	0.30 (0.06,1.43)	0.132
Ischaemic bowel following intervention: No	24 (92)	35 (43)	*	*
Ischaemic bowel following intervention: Yes	0 (0)	25 (31)	*	*
Ischaemic bowel following intervention: NA	2 (8)	22 (27)	*	*
ACS following intervention: No	23 (89)	30 (38)	*	*
ACS following intervention: Yes	0 (0)	9 (11)	*	*
ACS following intervention: NA	3 (12)	40 (51)	*	*
If ACS developed, abdomen re-opened: No	0 (0)	6 (60)	*	*

	Alive (n=27), n (%)	Dead (n=83), n (%)	Univariable analysis	
			OR (95% CI)	P value
If ACS developed, abdomen re-opened: Yes	0 (0)	3 (30)	*	*
If ACS developed, abdomen re-opened: NA	0 (0)	1 (10)	*	*
Central line sepsis: No	12 (46)	28 (35)	*	*
Central line sepsis: Yes	1 (4)	3 (4)	*	*
Central line sepsis: NA	13 (50)	48 (61)	*	*
**Hospital level variables**				
Paediatric surgeons/million children, mean (SD)	2.21 (0.83)	2.42 (0.74)	1.43 (0.62,3.28)	0.399
Personnel: low	0 (0)	2 (3)	*	*
Personnel: medium	20 (83)	66 (89)	*	*
Personnel: high	4 (17)	6 (8)	*	*
Infrastructure: low	1 (4)	9 (12)	reference	
Infrastructure: medium	11 (46)	34 (45)	0.33 (0.02,4.59)	0.408
Infrastructure: high	12 (50)	33 (43)	0.13 (0.01,2.32)	0.167
Procedure: low	8 (33)	42 (55)	reference	
Procedure: medium	7 (29)	25 (33)	0.70 (0.18,2.78)	0.610
Procedure: high	9 (38)	9 (12)	0.16 (0.03,0.84)	**0.031**
Anaesthesia and Resuscitation: low	4 (17)	5 (7)	reference	
Anaesthesia and Resuscitation: medium	14 (58)	51 (67)	2.77 (0.42,18.13)	0.289
Anaesthesia and Resuscitation: high	6 (25)	20 (26)	2.58 (0.34,19.75)	0.362

* Univariable analysis not possible due to low/zero counts or singularity of random intercept.
ACS: Abdominal compartment syndrome. ASA: American Association of Anesthesiologists. CI: Confidence interval. IQR: Interquartile range. NA: Not applicable. OR: Odds ratio. SD: Standard deviation. WHO: World Health Organisation.
Percentages have been rounded and may not total 100.

Appendix Table 7. Univariable analysis of patient-level and hospital-level factors associated with mortality for patients with ARM (generic and condition specific variables)

	Alive (n=167), n (%)	Dead (n=21), n (%)	Univariable analysis	
			OR (95% CI)	P value
**Patient level variables: *generic***				
Gestational age, median (IQR)	38 (37,38)	38 (37,38)	0.89 (0.66,1.20)	0.450
Male sex	88 (53)	15 (71)	2.70 (0.88,8.30)	0.082
Weight in kg, median (IQR)	3.4 (2.8,7.0)	2.9 (2.5,3.1)	0.64 (0.44,0.95)	**0.025**
Time to presentation in days, median (IQR)	5 (3,120)	3 (2,5)	0.94 (0.85,1.04)	0.251
Distance from home in km, median (IQR)	50 (25,150)	30 (14,59)	1.00 (0.99,1.00)	0.231
Mode of transport: Ambulance/ healthcare vehicle	28 (17)	3 (14)	reference	
Mode of transport: Patient's own	135 (83)	18 (86)	1.45 (0.31,6.83)	0.641
ASA score: 1-2	125 (82)	7 (35)	reference	
ASA score: 3-5	28 (18)	13 (65)	9.84 (2.75,35.28)	**<0.001**
WHO Checklist used: No	62 (37)	7 (33)	reference	
WHO Checklist used: Yes	85 (51)	8 (38)	0.69 (0.21,2.25)	0.540
WHO Checklist used: NA	20 (12)	6 (29)	1.06 (0.20,5.55)	0.941
Anaesthetic: General anaesthetic	140 (84)	12 (57)	reference	
Anaesthetic: No general anaesthetic	7 (4)	3 (14)	5.36 (1.00,28.90)	0.051
Anaesthetic: NA, no intervention	20 (12)	6 (29)	2.96 (0.82,10.76)	0.099
Anaesthetist: Doctor	106 (64)	8 (38)	reference	
Anaesthetist: Non-doctor	41 (25)	7 (33)	2.29 (0.66,7.98)	0.194
Anaesthetist: NA	20 (12)	6 (29)	3.20 (0.82,12.49)	0.095
Blood transfusion: Yes	26 (16)	10 (50)	5.53 (1.87,16.39)	**0.002**
Surgical site infection: Yes	34 (23)	3 (23)	1.06 (0.22,5.25)	0.940
Wound dehiscence: Yes	14 (10)	1 (8)	0.96 (0.09,10.50)	0.973
Further intervention: Yes	18 (12)	1 (7)	0.62 (0.07,5.60)	0.668
**Patient level variables: *condition specific***				
Type: High	79 (48)	15 (71)	2.71 (0.92,7.98)	0.070
No additional anomalies: Yes	126 (75)	10 (48)	0.24 (0.08,0.73)	**0.012**
Septic on arrival: Yes	28 (17)	13 (62)	8.55 (2.89,25.34)	**<0.001**
Antibiotics within an hour of arrival: Yes	52 (32)	8 (38)	1.42 (0.49,4.16)	0.521
Antibiotics for 48hours post-op: Yes	128 (83)	15 (79)	0.82 (0.20,3.41)	0.788
Ventilation available when required: No	48 (30)	12 (57)	reference	
Ventilation available when required: Yes	30 (19)	4 (19)	0.60 (0.15,2.40)	0.469
Ventilation available: NA, not required	83 (52)	5 (24)	0.24 (0.07,0.82)	**0.022**
Pre-op bowel perforation: Yes	6 (4)	1 (5)	1.63 (0.14,18.54)	0.692
Time to intervention in days, median (IQR)	2 (1,4)	2 (1,2)	0.81 (0.62,1.06)	0.127
Intervention: Stoma	100 (61)	13 (62)	reference	
Intervention: Anorectal reconstruction	44 (27)	2 (10)	0.35 (0.07,1.70)	0.194
Intervention: Other	19 (12)	2 (10)	0.72 (0.13,4.01)	0.704
Intervention: Palliative care	1 (1)	4 (19)	25.91 (2.21,303.3)	0.01
Pena stimulator used: Yes	32 (60)	2 (50)	*	*
Electrolyte disturbance: No†	124 (78)	4 (21)	reference	
Electrolyte disturbance: Yes†	15 (9)	11 (58)	29.94 (6.31,142.05)	**<0.001**
Electrolyte disturbance: NA†	21 (13)	4 (21)	5.82 (1.16,29.19)	**0.032**
High stoma output: No†	106 (65)	8 (40)	*	*
High stoma output: Yes†	4 (3)	0 (0)	*	*
High stoma output: NA†	52 (32)	12 (60)	*	*
Stoma prolapse: No†	105 (65)	8 (40)	reference	
Stoma prolapse: Yes†	4 (3)	1 (5)	4.02 (0.32,50.68)	0.282
Stoma prolapse: NA†	53 (33)	11 (55)	2.51 (0.85,7.46)	0.097
Peri-stoma skin breakdown: No†	87 (54)	8 (40)	reference	
Peri-stoma skin breakdown: Yes†	20 (12)	2 (10)	1.02 (0.18,5.88)	0.983
Peri-stoma skin breakdown: NA†	54 (34)	10 (50)	1.71 (0.54,5.43)	0.363
Sepsis: No†	126 (78)	6 (32)	reference	

	**Alive (n=167), n (%)**	**Dead (n=21), n (%)**	**Univariable analysis**	
			OR (95% CI)	P value
Sepsis: Yes†	12 (8)	10 (53)	22.20 (5.05,97.68)	**<0.001**
Sepsis: NA†	23 (14)	3 (16)	2.06 (0.37,11.59)	0.411
Anal stenosis: No†	77 (48)	5 (26)	ref	
Anal stenosis: Yes†	6 (4)	1 (5)	1.85 (0.14,25.12)	0.643
Anal stenosis: NA†	77 (48)	13 (68)	2.61 (0.76,9.00)	0.128
**Hospital level variables**				
Paediatric surgeons/million children, mean (SD)	2.13 (0.84)	2.00 (0.92)	1.03 (0.45,2.32)	0.953
Personnel: low	28 (18)	0 (0)	*	*
Personnel: medium	105 (69)	14 (74)	*	*
Personnel: high	20 (13)	5 (26)	*	*
Infrastructure: low	27 (17)	1 (5)	*	*
Infrastructure: medium	94 (58)	19 (95)	*	*
Infrastructure: high	41 (25)	0 (0)	*	*
Procedure: low	39 (24)	9 (45)	reference	
Procedure: medium	104 (64)	8 (40)	0.29 (0.07,1.21)	0.089
Procedure: high	19 (12)	3 (15)	0.58 (0.09,3.84)	0.574
Anaesthesia and Resuscitation: low	23 (14)	3 (15)	reference	
Anaesthesia and Resuscitation: medium	97 (60)	11 (55)	0.68 (0.10,4.65)	0.691
Anaesthesia and Resuscitation: high	42 (26)	6 (30)	0.83 (0.10,6.56)	0.857

* Univariable analysis not possible due to low/zero counts or singularity of random intercept. † Within 30-days of surgery. ASA: American Association of Anesthesiologists. CI: Confidence interval. IQR: Interquartile range. NA: Not applicable. OR: Odds ratio. SD: Standard deviation. WHO: World Health Organisation.

. *BMJ Global Health* 2021; 6:e004406. doi: 10.1136/bmjgh-2020-004406

Appendix Table 8. Univariable analysis of patient-level and hospital-level factors associated with mortality for patient with intussusception (generic and condition specific variables)

	Alive n=203, (n (%)	Dead (n=21), N (%)	Univariable analysis	
			OR (95% CI)	P value
**Patient level variables: *generic***				
Gestational age at birth, mean (SD)	39 (1)	38 (1)	0.74 (0.52,1.06)	0.103
Age at presentation in months, median (IQR)	7 [5, 10]	6 [5, 9]	0.98 (0.93,1.03)	0.418
Male sex	134 (66.0)	13 (61.9)	0.84 (0.32,2.20)	0.723
Weight in kg, median (IQR)	7.4 [6.4, 8.7]	8.0 [6.5, 8.0]	0.96 (0.83,1.11)	0.568
Onset to presentation in days, median (IQR)	3 [2, 5]	5 [4, 7]	1.00 (0.95,1.06)	0.897
Distance from home in km, median (IQR)	20 [10, 45]	23 [11, 50]	*	*
Mode of Transport: patient's own vs ambulance/health service vehicle	184 (91.5)	18 (85.7)	0.55 (0.14,2.24)	0.409
ASA score: 1-3	187 (94.9)	10 (47.6)	*	*
ASA score: 4-5	10 (5.1)	11 (52.4)	*	*
WHO Checklist used: No	111 (54.7)	13 (61.9)	reference	
WHO Checklist used: Yes	82 (40.4)	5 (23.8)	0.45 (0.13,1.55)	0.205
WHO Checklist used: NA, no intervention	10 (4.9)	3 (14.3)	3.03 (0.62,14.82)	0.170
Anaesthetic: General anaesthetic	155 (76.7)	16 (76.2)	reference	
Anaesthetic: No general anaesthetic	38 (18.8)	1 (4.8)	0.27 (0.03,2.22)	0.223
Anaesthetic: NA, no intervention	9 (4.5)	4 (19.0)	5.67 (1.23,26.21)	**0.026**
Anaesthetist: Doctor	121 (59.6)	13 (61.9)	reference	
Anaesthetist: Non-doctor	50 (24.6)	4 (19.0)	0.69 (0.18,2.60)	0.587
Anaesthetist: NA, no anaesthetic	32 (15.8)	4 (19.0)	1.50 (0.38,5.98)	0.563
Blood transfusion: Yes	72 (35.6)	16 (80.0)	7.97 (2.33,27.24)	**0.001**
Surgical site infection: Yes	43 (21.3)	2 (9.5)	0.37 (0.08,1.75)	0.211
Wound dehiscence: Yes	13 (6.4)	0 (0.0)	*	*
Further intervention: Yes	24 (11.8)	2 (9.5)	*	*
**Patient level variables: *condition specific***				
Diagnosis: clinical	35 (17.2)	5 (23.8)	reference	
Diagnosis: US	162 (79.8)	12 (57.1)	0.54 (0.17,1.76)	0.306
Diagnosis: other	6 (3.0)	4 (19.0)	5.16 (0.88,30.40)	0.070
Septic on arrival: Yes	82 (40.4)	19 (90.5)	14.59 (3.10,68.70)	**0.001**
Antibiotics given within 1hour of arrival: Yes	118 (58.1)	14 (66.7)	1.48 (0.53,4.15)	0.455
If hypovolaemic or septic: IVF given pre-intervention: No	20 (9.9)	0 (0.0)	*	*
If hypovolaemic or septic: IVF given pre-intervention: Yes	135 (66.8)	20 (95.2)	*	*
If hypovolaemic or septic: IVF given pre-intervention: N/A	47 (23.3)	1 (4.8)	*	*
Peritonitis: Yes†	34 (16.7)	7 (33.3)	2.80 (0.93,8.40)	0.067
Perforation: Yes†	9 (4.4)	6 (28.6)	*	*
Shock: Yes†	7 (3.4)	5 (23.8)	8.98 (2.21,36.50)	**0.002**
Other contraindication: Yes†	54 (26.6)	4 (19.0)	0.61 (0.18,2.01)	0.414
No contraindication†	104 (51.2)	3 (14.3)	*	*
Intervention: laparotomy vs air or hydroenema reduction	140 (70.7)	17 (100.0)	*	*
Successful air or hydroenema reduction: Yes	40 (69.0)	0 (0.0)	*	*
Management at laparotomy: manual reduction	63 (39.6)	0 (0.0)	*	*
Management at laparotomy: resection, primary anastomosis	93 (58.5)	14 (82.4)	*	*
Management at laparotomy: resection, stoma	3 (1.9)	3 (17.6)	*	*
Time from onset to reduction/management in hours, median IQR	15 [6,24]	20 [9, 24]	1.00 (0.99,1.01)	0.848
Recurrence: Yes	3 (1.5)	1 (5.0)	*	*

. BMJ Global Health 2021; 6:e004406. doi: 10.1136/bmjgh-2020-004406

	**Alive n=203, (n (%)**	**Dead (n=21), N (%)**	**Univariable analysis**	
			OR (95% CI)	P value
**Hospital level variables†**				
Paediatric surgeons/million children, mean (SD)	2.24 (0.84)	2.05 (0.94)	0.79 (0.42,1.50)	0.474
Personnel: low	28 (15.7)	1 (5.0)	reference	
Personnel: medium	130 (73.0)	16 (80.0)	3.84 (0.37,39.36)	0.257
Personnel: high	20 (11.2)	3 (15.0)	4.72 (0.34,66.28)	0.250
Infrastructure: low	28 (14.0)	1 (4.8)	reference	
Infrastructure: medium	135 (67.5)	17 (81.0)	4.23 (0.41,44.13)	0.227
Infrastructure: high	37 (18.5)	3 (14.3)	2.62 (0.19,35.51)	0.469
Procedure: low	34 (17.0)	6 (28.6)	reference	
Procedure: medium	157 (78.5)	14 (66.7)	0.51 (0.16,1.56)	0.237
Procedure: high	9 (4.5)	1 (4.8)	0.60 (0.06,6.35)	0.674
Anaesthetic and resuscitation: low	27 (13.5)	3 (14.3)	reference	
Anaesthetic and resuscitation: medium	112 (56.0)	11 (52.4)	0.91 (0.18,4.53)	0.904
Anaesthetic and resuscitation: high	61 (30.5)	7 (33.3)	1.02 (0.18,5.62)	0.984

* Univariable analysis not possible due to low/zero counts or singularity of random intercept. † As a contra-indication for air- or hydro-enema reduction. ASA: American Association of Anesthesiologists. CI: Confidence interval. IQR: Interquartile range. IVF: Intravenous fluid. NA: Not applicable. OR: Odds ratio. SD: Standard deviation. WHO: World Health Organisation. US: Ultrasound. Percentages have been rounded and may not total 100.

Appendix Table 9. Patient data validation: agreement between the study dataset and validation dataset

Variable	Observed agreement % (patients with agreement/ total patients)*	Kappa statistic†
**Generic variables:**		
Sex	90% (45/50)	0.80
Did the patient survive to discharge	98% (49/50)	0.85
Did the patient require a further intervention	96% (43/45)	-
Study condition	100% (50/50)	1.00
**Condition specific variables:**		
Gestational age at birth (in patients with gastroschisis and anorectal malformation)	83% (5/6)	0.85
Type of gastroschisis - simple	100% (5/5)	1.00
Type of gastroschisis - associated with atresia	80% (4/5)	-
Type of gastroschisis - associated with necrosis	80% (4/5)	0.55
Type of gastroschisis - associated with perforation	100% (5/5)	1.00
Method of gastroschisis closure‡	60% (3/5)	0.44
Type of anorectal malformation	50% (1/2)	-
Anorectal malformation primary operation	100% (2/2)	1.0
Age at presentation in patients with appendicitis	94% (17/18)	0.98
Management of appendicitis§	94% (17/18)	0.79
Operative findings in those with appendicitis	100% (18/18)	1.00
Age at presentation in patients with intussusception	60% (3/5)	0.41
Was the intussusception diagnosed on ultrasound	100% (5/5)	1.00
Primary management in those with intussusception**	60% (3/5)	-
Age at diagnosis (in weeks) in patients with an inguinal hernia	90% (18/20)	0.94
Operation type (emergency or elective) in patients with an inguinal hernia	95% (19/20)	-
Operation undertaken in patients with an inguinal hernia	100% (20/20)	1.00
**Total**	**94% (336/359)**	**Median: 0.96**

* Data from 50 patients in 3 hospitals; 2 hospitals randomly selected for validation could not participate due to unavailability of the required data retrospectively. † 0.60-0.80 is good agreement; 0.81-1 is very good agreement. For some variables the kappa statistic could not be calculated due to all data confined to one category. ‡ Two differences were between preformed and surgical silo. § Difference was 'other' category. ** Main dataset had air enema reduction as primary intervention for two patients and the validation dataset had laparotomy – one explanation might be the validation data was collected from the operating room logbook retrospectively and hence the prior air enema reductions undertaken outside the OR were missed.

Appendix Table 10. Validation survey undertaken by local investigators at validating centers

Survey Question	No. of local investigators who collected study data (n=8), n (%)	No. of local investigators who collected validation data (n=4), n (%)
**Do you think your team managed to identify all eligible patients for the study?**		
Yes	8 (100)	3 (75)
No	0 (0)	0 (0)
Unsure	0 (0)	1 (25)
**Could any of the patients have been missed from study inclusion?**		
Yes	3 (38)	1 (25)
No	4 (50)	3 (75)
Unsure	1 (13)	0 (0)
**If yes or unsure, how might patients have been missed from study inclusion?***		
Patients who are discharged against medical advice or died before paediatric surgery team review.	2	1
The resident collecting data may have missed a patient, especially if the data was collected after the patient's intervention.	1	0
On nights and weekends the adult surgeons may also manage pediatric patients. Although general surgery daily service lists were checked, there is a possibility that one or two patients could be missed.	1	0
Poor record keeping.	0	1
**Are there any conditions that were more likely to be missed from study inclusion?**		
Gastroschisis	1 (13)	1 (25)
Anorectal malformation	0 (0)	0 (0)
Appendicitis	2 (25)	2 (50)
Intussusception	0 (0)	0 (0)
Inguinal hernia	5 (63)	1 (25)
**If any of the conditions were more likely to be missed, why was this the case?***		
Inguinal hernia – may be seen in out-patients only, often managed as day cases, there are lots of patients with this condition.	3	1
Appendicitis – some of the older children may have been managed by adult general surgeons.	2	1
Gastroschisis – more likely to discharge against medical advice than other cases.	1	0
**Which patients with appendicitis did you include in the study?**		
Just patients within the paediatric services.	4 (50)	4 (75)
All eligible patients from adult and paediatric services.	4 (50)	1 (25)
**How did you identify patients to include in the study?**		
Ward round	6 (75)	0 (0)
Handover	5 (63)	0 (0)
Operating room logbook	4 (50)	3 (75)
Planned operation lists	4 (50)	1 (25)
Ward patient lists	8 (100)	4 (100)

Word of mouth	2 (25)	0 (0)
Personal knowledge of patients	5 (63)	0 (0)
Other	1 (13)	1 (25)
**If none of the collaborators were present at the hospital for one or more days during the study, was the team able to identify patients eligible for the study on those days?**		
Yes	6 (75)	3 (75)
No	0 (0)	0 (0)
Not applicable	2 (25)	0 (0)
Unsure	0 (0)	1 (25)
**How did you identify patients to be included in the study on days when study collaborators were not present at the hospital?***		
Handover	2	0
Operating room logbook	1	2
Ward patient lists	2	0
Word of mouth	3	0
Personal knowledge of patients	1	0
Not applicable – there is never a time when a member of the pediatric surgery team is not on the ground.	3	0
Emergency records	1	0
Case logs/ ward book/ electronic medical record system for billing	1	3
**Do you have any concerns regarding the accuracy of the data collected?**		
Yes	2 (25)	1 (25)
No	5 (62.5)	3 (75)
Unsure	1 (12.5)	0 (0)
**What data points might be inaccurate and what were the challenges for collecting the data?***		
Collecting exact distances from home to hospital.	2	0
Accuracy of gestational age stated by illiterate mothers.	2	0
Exact duration of time between presentation and primary intervention – these may not be documented.	2	0
Poor record keeping.	0	1
**Were any of the data points more difficult to collect accurately? If so, which ones and why?***		
Following up patients managed by the general adult surgeons to 30-days following intervention.	1	0
Distance from home to hospital. Most cities are not planned and distances are not accurately stated. We used Google distance calculator in some cases and estimated some. Some patients come from ‘these parts’ and do not have an accurate name for calculating. People measure distance of travel by cost of the transport.	3	0
Gestational age – illiterate mothers often estimate pregnancy in months rather than weeks. Many do not have an ultrasound for dating. Records say ‘full term baby’ which is anything from 37 to 40 weeks.	2	2
Time from admission to intervention. Paediatricians may review patients first and not document the time of admission. Some cases had no timing documented on the anesthetic chart.	2	0

* Free text boxes: percentages not calculated. Percentages have been rounded and may not total 100.

Appendix Table 11. Validation of the hospital data (n=40)

Variable	Observed agreement*, % (no. hospitals with agreement/ total no. validation hospitals)	ICC†
Personnel		
Population served by the hospital in millions	23% (5/22)	1.00
No. Paediatric Surgeons undertaking general paediatric surgery per hospital	74% (28/38)	0.87
No. Paediatric Surgeons undertaking neonatal surgery per hospital	63% (24/38)	0.86
No. General Surgeons undertaking general paediatric surgery per hospital	58% (22/38)	0.80
No. General Surgeons undertaking neonatal surgery per hospital	68% (26/38)	0.71
No. Medical Officers undertaking paediatric surgery per hospital	68% (25/37)	0.48
No. Medical Officers undertaking neonatal surgery per hospital	81% (30/37)	0.06
Infrastructure and surgical resources		
Running water	71% (27/38)	0.55
Electricity	63% (24/38)	0.50
Electricity generator back-up	74% (28/38)	0.44
Laboratory for biochemistry	76% (29/38)	0.53
Laboratory for hematology	71% (27/38)	0.32
Blood bank	62% (25/40)	0.12
Functioning ultrasound (US) machine	72% (29/40)	0.38
Fluoroscopy	78% (31/40)	0.58
Paediatric ventilation outside of the operating room (OR)	85% (34/40)	0.88
Neonatal ventilation outside of the OR	90% (36/40)	0.93
Availability of a Paediatric Intensive Care Unit (ICU) for surgical patients	87% (33/38)	0.67
Availability of Neonatal ICU for surgical patients	84% (32/38)	0.67
Parenteral nutrition	90% (36/40)	0.81
Surgical Safety Checklist in the OR	62% (25/40)	0.60
Sterile gloves and gown	84% (32/38)	-
Autoclave for sterilising surgical equipment	87% (33/38)	-
Pena stimulator for identifying the muscle complex in ARM surgery	74% (28/38)	0.67
Procedures		
Cotside reduction and primary closure of gastroschisis (Bianchi technique)	95% (37/39)	0.72
Preformed silo application, reduction and closure of gastroschisis	79% (31/39)	0.6
Surgical silo application, reduction and closure of gastroschisis	59% (23/39)	0.35
Primary closure of gastroschisis in the OR	64% (25/39)	0.48
Sigmoid colostomy for anorectal malformation	75% (30/40)	0.38
Posterior sagittal anorectoplasty (PSARP) for anorectal malformation	78% (31/40)	0.49
Open appendicectomy	85% (34/40)	-
Laparoscopic appendicectomy	80% (32/40)	0.56
US drainage of intra-abdominal collection	70% (26/37)	0.57
CT drainage of intra-abdominal collection	84% (32/38)	0.34
US diagnosis of intussusception	78% (31/40)	0.54
Air-enema reduction of intussusception	92% (36/39)	0.93
Hydro-enema reduction of intussusception	87% (34/39)	0.68
Laparotomy for intussusception	62% (25/40)	-
Open inguinal herniotomy	98% (39/40)	0.61
Laparoscopic inguinal herniotomy	90% (35/39)	0.67
Paediatric central line insertion	75% (30/40)	0.56
Neonatal central line insertion	82% (33/40)	0.70
Umbilical catheterisation	72% (28/39)	0.69
Anaesthesia and resuscitation		
Paediatric bag, value and mask	70% (28/40)	0.14
Bottled oxygen	75% (30/40)	0.40
Piped oxygen	82% (33/40)	0.60
Oxygen saturation monitor	72% (29/40)	0.27
Apnoea monitor	71% (27/38)	0.61
Multi-parameter intra-operative monitoring	66% (25/38)	0.02

**Variable**	**Observed agreement*, % (no. hospitals with agreement/ total no. validation hospitals)**	**ICC†**
Anaesthetic machine for children	60% (24/40)	0.15
Anaesthetic machine for neonates	68% (27/40)	0.46
Ketamine anaesthesia for children	65% (26/40)	0.22
Ketamine anaesthesia for neonates	68% (26/38)	0.22
Spinal/ caudal anaesthesia for children	80% (32/40)	0.54
Spinal/ caudal anaesthesia for neonates	84% (32/38)	0.58
Anaesthetic doctor competent to perform paediatric anaesthesia	85% (34/40)	0.75
Anaesthetic doctor competent to perform neonatal anaesthesia	82% (33/40)	0.71
Anaesthetic nurse competent to perform paediatric anaesthesia	68% (27/40)	0.61
Anaesthetic nurse competent to perform neonatal anaesthesia	75% (30/40)	0.61
Presence of a children's hospital within the country of the participating hospital	70% (28/40)	0.58
Total	75% (1752/2327)	Median 0.58, IQR 0.41,0.69

* Observed agreement (always vs sometimes/never) between the two most senior team members who completed the survey at each hospital. Only hospitals with more than one rater were included (n=40).
† Interclass Correlation Coefficients (ICCs) were calculated from mixed models with hospital as the random intercept. ICC interpretation: <0.40 poor, 0.40-0.59 fair, 0.60-0.74 good, 0.75-1.00 excellent. Results represent the level of agreement between all respondents from each hospital where more than one respondent completed the survey. For some variables the ICC could not be calculated due to all data confined to one category.

. BMJ Global Health 2021; 6:e004406. doi: 10.1136/bmjgh-2020-004406
